# Understanding the role and organization of health workers delivering non-communicable disease management in primary care in low- and middle-income countries: a scoping review

**DOI:** 10.1186/s12875-025-03033-3

**Published:** 2025-11-17

**Authors:** Archna Gupta, Oluwasegun J. Ogundele, Roxana Rabet, Iryna Artyukh, Thiago Trindade, Doret Cheng, Daniel Osafo Darko, Mai Eltigany, Alarcos Cieza, Becky Skidmore, Katherine D. Rouleau

**Affiliations:** 1 Upstream Lab, MAP Centre for Urban Health Solutions, Li Ka Shing Knowledge Institute, Unity Health Toronto, 30 Bond Street, Toronto, ON M5B 1W8 Canada; 2 Department of Family and Community Medicine, St. Michael’s Hospital, Toronto, ON Canada; 3 Department of Family and Community Medicine, University of Toronto, Toronto, ON Canada; 4 Independent Information Specialist, Ottawa, ON Canada; 5 Nyaho Medical Centre, Accra, Ghana; 6 Central University, Miotso, Ghana; 7 Department of Clinical Medicine, Federal University of Rio Grande Do Norte, Natal, Brazil; 8 Department of Noncommunicable Diseases and Department of Rehabilitation and Disability, World Health Organization, Geneva, Switzerland

**Keywords:** Primary Health Care, Non-Communicable Diseases, Mental Health, Multidisciplinary teams, Models of Care

## Abstract

**Background:**

Non-communicable diseases are responsible for three-fourths of annual deaths worldwide and disproportionately affect individuals living in low- and middle-income countries. As populations age and the burden of chronic diseases rises, the role of the health workforce becomes increasingly vital in ensuring equitable access to prevention, early detection, and appropriate treatment. This study aims to review and synthesize the existing knowledge on the role and organization of health workers in managing non-communicable diseases in primary care in low- and middle-income countries.

**Methods:**

We followed the PRISMA-SCR guidelines and conducted a scoping review in the MEDLINE, EMBASE, CINAHL, and Global Index Medicus databases. We included studies that addressed a non-communicable disease, specified the health workers involved, and reported on models of care for chronic disease management. These studies were published in English.

**Results:**

We identified 175 articles. One hundred twenty-five (71%) highlighted the role of multidisciplinary teams, and 41 (23%) discussed optimizing roles within teams for the management of non-communicable diseases in primary care. Multidisciplinary teams often included generalist physicians, nurses, and community health workers. Optimizing roles within teams involves redistributing tasks from doctors to nurses or community health workers and from nurses to community health workers.

**Conclusions:**

Multidisciplinary teams and optimizing health workers' roles within teams are important in delivering non-communicable disease management. Multidisciplinary teams typically included generalist physicians, nurses, and community health workers, while additional non-physician health workers depended on the most prevalent health conditions being addressed in the community served. Optimizing the roles of health workers provides opportunities to redistribute responsibilities to deliver more comprehensive care.

**Supplementary Information:**

The online version contains supplementary material available at 10.1186/s12875-025-03033-3.

## Background

The management of non-communicable diseases (NCDs) in primary care in low- and middle-income countries (LMICs) is a pressing global health challenge[1–3].NCDs are responsible for approximately 75% of all annual non-pandemic-related deaths worldwide[4]. Seventeen million yearly NCD-related deaths occur prematurely, before the age of 70, of which 86% occur in LMICs[5]. In all regions of the world, individuals in lower socio-economic quintiles face a disproportionately higher risk of morbidity and mortality due to NCDs[4].

The management of NCDs presents unique challenges for health systems. NCDs typically develop over a lifetime, often remaining undetected for years before symptoms appear[3]. Their chronic nature demands lifelong care, requiring patients to actively engage with the healthcare system to manage and restore their health[6].Managing NCDs sometimes involves complex medication regimens as well as behavioural changes influenced by psychological, social, commercial, and environmental determinants, making their treatment multifaceted and long-term[4, 7].

In Primary Health Care (PHC)-oriented health systems, health services are integrated with primary care and essential public health functions, including health promotion and prevention, at their core[8]. Furthermore, despite challenges, opportunities for synergy in managing NCDs exist. Many NCDs share common risk factors and behavioural drivers, presenting an opportunity for integrated care across the entire health spectrum – from health promotion and disease prevention to acute and chronic care, rehabilitation, and palliative care[9]. To achieve this, person-centred care delivered by an accessible and collaborative health workforce is essential[9].

In LMICs, an integrated health services approach has often involved “adding” specific NCD-focused services to existing vertical programs, such as those targeting HIV or maternal and child health[10–12]. While these programs have achieved undeniable success, services are not always anchored to primary care or organized to make comprehensive, holistic, and continuous care universally available to the whole population. A more sustainable and possibly effective solution lies in models of care that integrate NCD-related services within primary care, ensuring comprehensive care across the spectrum of care.

High-quality primary care is one component of PHC-oriented health systems, and is defined by several key characteristics, including reliable first-contact access, continuity, comprehensiveness, coordination, and person-centeredness[13]. Primary care services are further delivered close to where people live and work and are informed by the needs and demands of the population. As such, PHC-oriented, high-quality primary care is optimally positioned as the foundation for tackling the multifaceted health challenges posed by NCDs and involves a health workforce able to deliver integrated care and services[14, 15].

Primary care services must be delivered through models of care that are explicitly oriented towards the PHC approach to achieve the desired objectives of better health outcomes, equity, and value. Models of care refer to “a conceptualization of how services should be delivered, including the processes of care, organization of providers, and management of services, supported by the identification of roles and responsibilities of different platforms and providers along the pathways of care.”[16] These models outline which health workers deliver what services, to which populations, in what settings, and using which strategies, processes, and tools.

In PHC-oriented health systems, then, NCD-related health needs are addressed through models of care that purposefully integrate services “around the whole person” in the community and within primary care as much as possible, across the full spectrum of services from promotion to prevention, to treatment, rehabilitation, and palliation, and, when necessary, that outline clear care pathways across levels.

The WHO Primary Health Care Measurement Framework and Indicators (PHCMFI) identifies four key “domains” of models of care: service packages, service design, organization and facility management, and community linkages and engagement[17]. These domains provide a foundation for evaluating the literature on models of care in delivering NCD services in primary care[17]. In practice, however, the success of any given model of care depends mainly on the resources invested and the effective organization of the health workforce within these models[17].That is to say that while several PHC levers are involved, the ultimate “active ingredients” of models of care are the individuals who deliver the care, the health workforce[17]. Because of its apparent human dimension, the health workforce is also the most challenging to change[18–21].

The health workforce plays a uniquely crucial role as it is not only the nexus of “care,” that is, the human interface between people and the health system, the locus of competency and a key driver of quality, but it also consistently receives a large portion of health budgets and, unlike “material resources,” cannot be entirely nor easily controlled[17, 22].Yet, in theory, the health workforce is one of the adaptable and responsive elements of health systems and, therefore, one that is relied upon to implement integration[17, 22].That is to say that the integration of services is ultimately operationalized through the health workforce, in the ways single health workers and teams or networks of health workers are trained and organized to deliver integrated services[9, 17, 22].

The health workforce is the backbone of healthcare delivery. Because primary care is the core of PHC-oriented health systems[23], the primary care workforce is crucial to highly performing health systems, including delivering NCD-related care and services through multidisciplinary teams (MDTs) providing integrated care. In LMICs where the health workforce is often stretched thin, especially in primary care, understanding how to optimize its role in managing NCDs is critical. The MDT approach, which encompasses coordinated planning, decision-making, and information sharing between different health providers, is particularly essential[24–26].

Despite the availability of guidance on addressing NCDs provided by the World Health Organization’s Package of Essential NCD interventions, significant gaps remain in the ability of primary care systems to deliver comprehensive care in LMICs[27–29]. This gap highlights the misalignment between primary care's intended role and its capacity to meet the complex needs of individuals affected by NCDs. Closing these gaps requires models of care that prioritize the health workforce's ability to deliver comprehensive, integrated, person-centred services. While previous reviews have explored integrated models of care to address the growing burden of NCDs in LMICs[30–36], few have focused specifically on the role of the health workforce in this context. A more nuanced understanding of how health workers contribute to NCD management in primary care is needed. As such, this review sought to understand what existing knowledge is available on the role of health workers in managing NCDs in primary care, an integral component of primary health care-oriented systems, in low- and middle-income countries.

## Methodology

We used a scoping review methodology. The study followed the Guidance for Conducting a Systematic Scoping Review[37]. A scoping review was chosen because it brings together emerging evidence and identifies gaps in the research knowledge base. The review protocol was registered on Open Science Framework (available from https://osf.io/nfm4u).

### Eligibility criteria

We identified studies that focused on models of care for NCDs within primary care in LMICs. We included studies that met the following criteria: (1) address NCDs in clinical or non-clinical contexts; (2) specify the health worker involved in the provision of care; (3) investigate, assess, or report on how models of care integrate the delivery of NCD-related services within primary care in LMICs; and (3) were published in English. Further details on the eligibility criteria are available online as Additional File 1: Appendix M1. We included prospective and retrospective studies, case–control studies, and cross-sectional studies. Additionally, we consulted the reference lists of systematic reviews and scoping studies to identify additional relevant studies. We excluded study protocols, commentaries, studies conducted in high-income countries, editorials, case reports, and conference abstracts. We identified non-English studies in the search; however, due to resource and time constraints, they are not included in this review.

### Information sources and search strategy

The literature review was conducted in two phases. During the first phase, a medical information specialist developed and tested the search strategies through an iterative process in consultation with the review team. Another senior information specialist peer-reviewed the MEDLINE strategy before execution using the PRESS Checklist[38]. Using the multifile option and the deduplication tool available on the Ovid platform, we searched Ovid MEDLINE® ALL and Embase. We also searched CINAHL (Ebsco) and the regional databases of Global Index Medicus (GIM). We performed all searches on January 13, 2024.

The search strategies utilized a combination of controlled vocabulary (e.g., “Health Personnel”, “Non-communicable Diseases”, “Delivery of Health Care, Integrated”, “Primary Health Care or Primary Care”) and keywords (e.g., “nurse”, “cancer”, “collaborative care”). We used both Primary Health Care and Primary Care in our search, recognizing that these terms are often used interchangeably despite having distinct definitions. We applied the Cochrane EPOC filter (December 2022 version) for LMICs to the MEDLINE, Embase Classic + Embase, and CINAHL searches. Vocabulary and syntax were adjusted across the databases, and where possible, animal-only records, opinion pieces, conferences, and case studies were removed. There were no language or date restrictions on any of the searches. Due to the multiple searches necessary for GIM, these records were downloaded separately and de-duplicated within themselves before combining them with the results of the other searches. During the second phase, additional articles were identified via hand-searching of relevant literature reviews retrieved in phase one and included for screening. All records were downloaded and de-duplicated using EndNote version 9.3.3 (Clarivate Analytics). All references were imported into Covidence to manage the review process. The complete search strategy is available online as Additional File 2: Appendix S1.

### Study selection

We conducted a two-stage screening process: a review of titles and abstracts and a full-text review. To ensure consistency among reviewers, study authors were trained in applying inclusion and exclusion criteria by screening a sample of 20 titles and abstracts. Nine independent reviewers were involved (IA, RR, SB, DC, DD, OO, TT, OW and MF). Two reviewers independently evaluated the titles and abstracts of all retrieved citations against predefined inclusion criteria. Articles identified as relevant by one or both reviewers proceeded to the full-text review. In the second stage, two reviewers independently assessed the full-text articles to determine their adherence to the inclusion and exclusion criteria. A third reviewer (AG) resolved any disagreements in the full-text evaluations. Articles deemed to be review articles at the second stage were screened separately. Reference lists were reviewed to identify if any additional papers were relevant and were added to the screening process. Regular meetings with the reviewers were held at the onset and midpoint of the review process to address challenges and clarify uncertainties.

### Data charting

The study team developed a data collection instrument designed explicitly to extract information from the studies included in this research. Collaboratively, the team formulated the data charting form, outlining the variables and the depth of information to be extracted. The data collection instrument was developed using the WHO Primary Health Care Measurement Framework and Indicators (PHCMFI). Elements from this framework that aligned with the health workforce were used to develop our data collection instrument, including variables from service packages, service design, organization and facility management, community linkages, engagement, and service delivery outputs[17].

To validate the instrument's reliability in capturing the information of interest and ensuring consistency within the team, a calibration exercise was conducted on ten randomly selected articles before the charting phases. Extracted variables included the year of publication, citation, country in which the study was conducted, study setting, study aims, intervention description, description of health workers delivering care, Health conditions described (e.g., NCDs, Mental health), task shifting or sharing, MDTs, population outreach, results of the model of care if included. The data charting form, including the variables collected, is available online as Additional File 3: Appendix S2.

Eight reviewers (IA, RR, SB, DC, DD, OO, TT, and MF) performed the data abstraction, and the abstracted data was compared for accuracy by the first author (AG). Quality assessments were not undertaken and were beyond the scope of this review[39, 40].

### Analysis

The review team comprised family physicians (AG, TT, DD, KR), pharmacists (DC), and LMIC primary care experts (DD and TT). The team assembled for a half-day data analysis workshop where the data was reviewed, emerging themes were discussed, and collectively, central themes which aligned with the PHCMFI framework were identified, which best addressed our research objectives. In this manuscript, we report on the most relevant variables to these themes.

Following Arksey and O’Malley’s guidelines for enhancing the rigour of a scoping review through consultations[41], we engaged with the WHO team (ME, AC, KR) at multiple stages of the process. This included input on developing the search strategy and data collection instruments, and consultation with the broader WHO team during the interpretation of results.

## Results

**Fig. 1:**
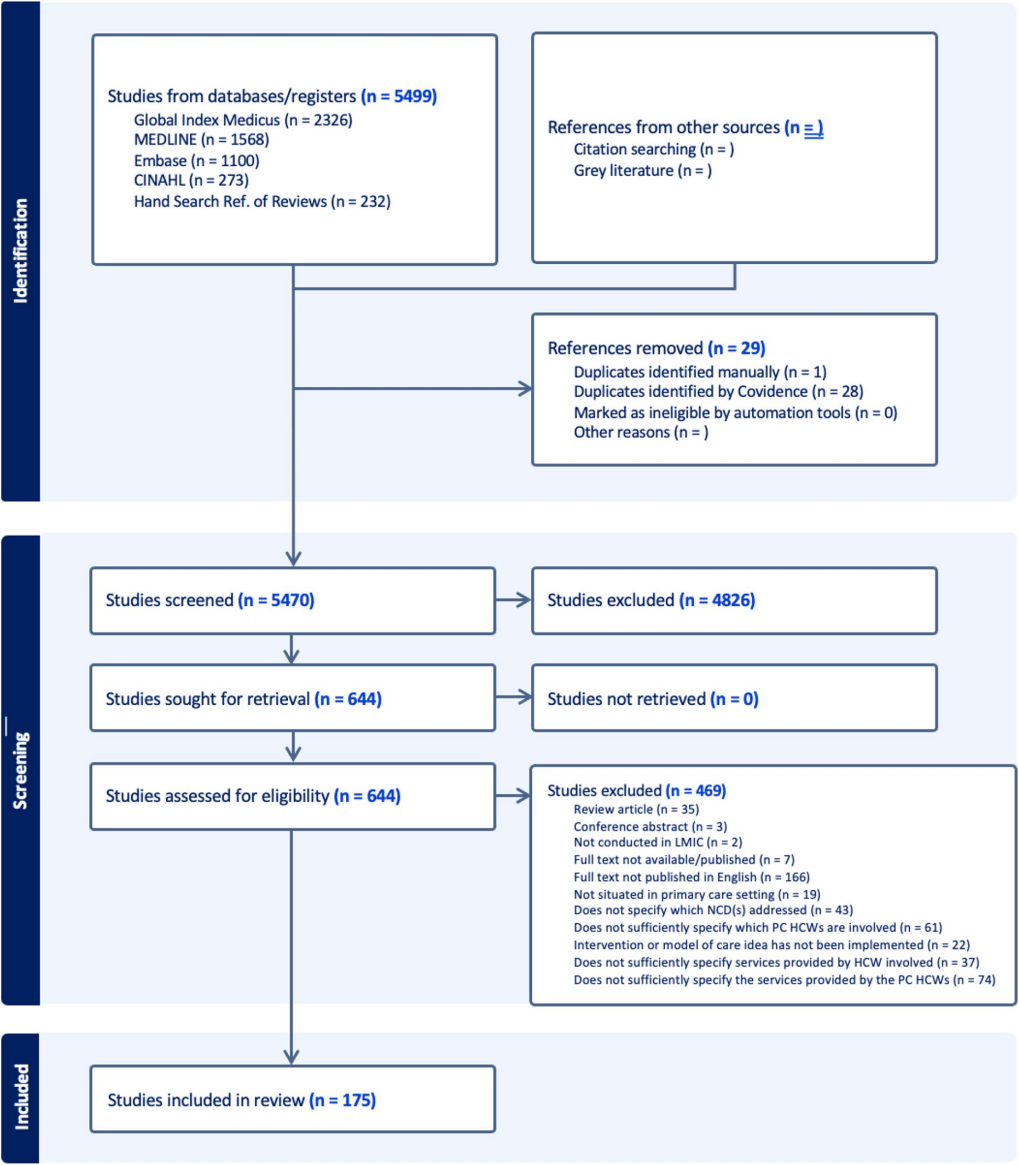
PRISMA diagram

We conducted a comprehensive search following PRISMA-ScR guidelines[42]. Our initial search yielded 5,499 studies, including 232 references from a manual review of reference lists of systematic and scoping review studies (Fig. 1). After removing duplicates, 5,470 peer-reviewed studies were screened based on their abstracts and titles, yielding 644 studies for full-text review. Of those, 469 did not meet our eligibility criteria and 175 articles were included in our analysis. A list of non-English papers identified during the search is provided in Additional File 4: Appendix S3.

### Characteristics

**Fig. 2:**
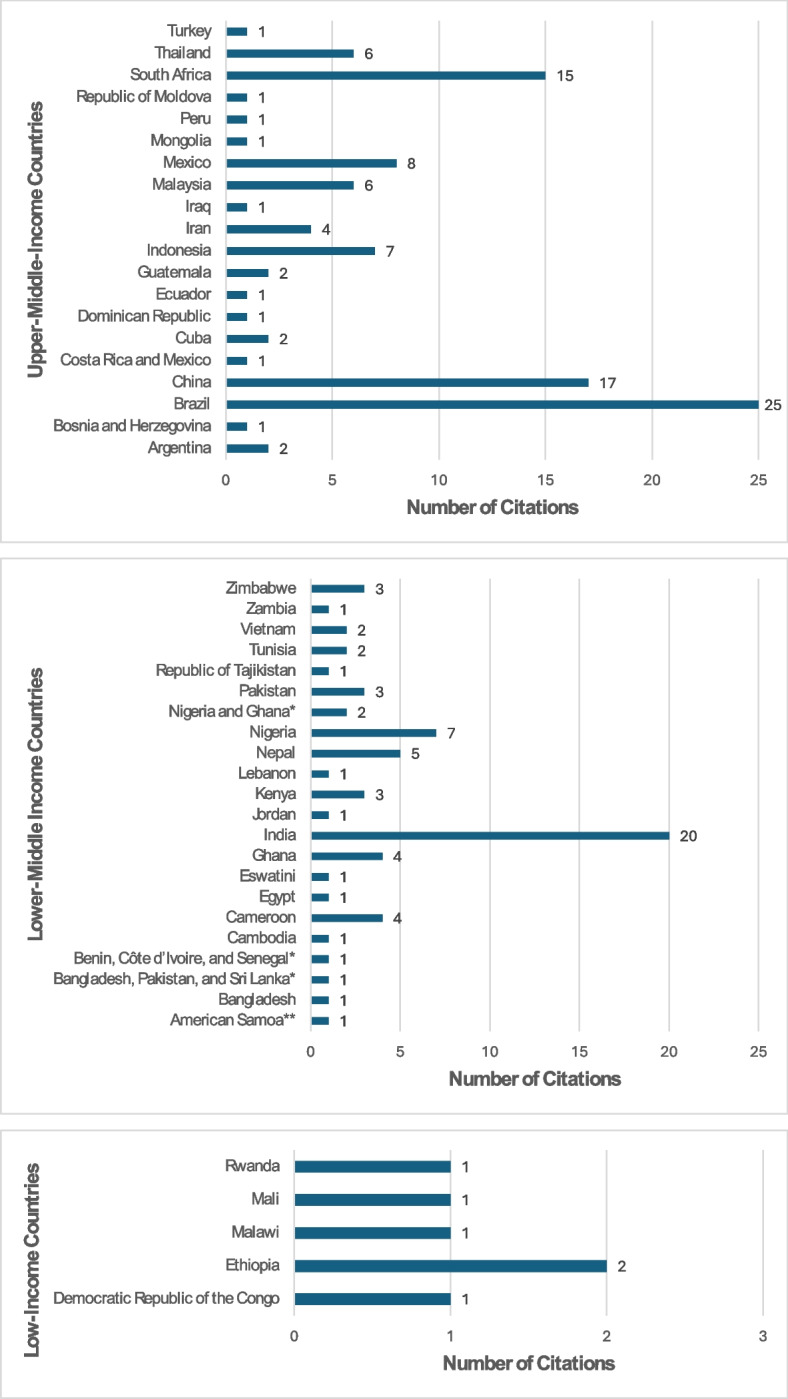
Distribution of citations by country income status

Table 1 and Fig. 2summarize studies by country, categorized based on WHO regions and World Bank income classification[43, 44]. The data illustrate various citations across different regions and income levels. Of the 175 studies included, five reported on multiple countries, and 170 reported on 42 individual countries. Upper-middle-income countries accounted for 59% of studies, with slightly more than half of those situated in Brazil (25 articles), China (17), and South Africa (15). Lower-middle-income countries made up 38% of reviewed studies, with studies from India (20 studies ), Nepal (5), and Nigeria (7) accounting for 44%. Low-income countries (3%), predominantly from the African region, accounted for fewer studies. Most studies come from the African (27%) and the Americas Regions (25%). The South-East Asian Region and Western Pacific Region each represent 19% of studies. Additional File 5: Appendix S4-S6 summarizes citations related to the distribution of study countries by WHO region and income classification.

**Table 1:** Citations by country, categorized by WHO region and income classification

Study Country	WHO Region	Income Classification	# of Study Citations	Reference
American Samoa**	WPRO	lower-middle-income	1	[60]
Argentina	AMRO	upper-middle-income	2	[122, 123]
Bangladesh	SEARO	lower-middle-income	1	[96, 124]
Bangladesh, Pakistan, and Sri Lanka*	SEARO	lower-middle-income	1	[96]
Benin, Côte d’Ivoire, and Senegal*	AFRO	lower-middle-income	1	[125]
Bosnia and Herzegovina	EURO	upper-middle-income	1	[126]
Brazil	AMRO	upper-middle-income	25	[51, 52, 80, 86, 127–148]
Cambodia	WPRO	lower-middle-income	1	[149]
Cameroon	AFRO	lower-middle-income	4	[58, 64, 65, 67]
China	WPRO	upper-middle-income	17	[48, 50, 81, 82, 85, 87, 95, 150–159]
Costa Rica and Mexico	AMRO	upper-middle-income	1	[160]
Cuba	AMRO	upper-middle-income	2	[161, 162]
Democratic Republic of the Congo	AFRO	low-income	1	[163]
Dominican Republic	AMRO	upper-middle-income	1	[76]
Ecuador	AMRO	upper-middle-income	1	[164]
Egypt	EMRO	lower-middle-income	1	[165]
Eswatini	AFRO	lower-middle-income	1	[63]
Ethiopia	AFRO	low-income	2	[73, 166]
Ghana	AFRO	lower-middle-income	4	[66, 68, 71, 167]
Guatemala	AMRO	upper-middle-income	2	[79, 168]
India	SEARO	lower-middle-income	20	[47, 70, 169–186]
Indonesia	SEARO	upper-middle-income	7	[94, 187–192]
Iran	EMRO	upper-middle-income	4	[83, 193–195]
Iraq	EMRO	upper-middle-income	1	[196]
Jordan	EMRO	lower-middle-income	1	[197]
Kenya	AFRO	lower-middle-income	3	[62, 198, 199]
Lebanon	EMRO	lower-middle-income	1	[200]
Malawi	AFRO	low-income	1	[74]
Malaysia	WPRO	upper-middle-income	6	[201–206]
Mali	AFRO	low-income	1	[45]
Mexico	AMRO	upper-middle-income	8	[49, 93, 207–212]
Mongolia	WPRO	upper-middle-income	1	[213]
Nepal	SEARO	lower-middle-income	5	[61, 214–217]
Nigeria	AFRO	lower-middle-income	7	[72, 218–222]
Nigeria and Ghana*	AFRO	lower-middle-income	2	[223, 224]
Pakistan	EMRO	lower-middle-income	3	[59, 225, 226]
Peru	AMRO	upper-middle-income	1	[227]
Republic of Moldova	EURO	upper-middle-income	1	[228]
Republic of Tajikistan	EURO	lower-middle-income	1	[229]
Rwanda	AFRO	low-income	1	[46]
South Africa	AFRO	upper-middle-income	15	[77, 78, 84, 88, 89, 91, 92, 230–237]
Thailand	WPRO	upper-middle-income	6	[75, 80, 90, 97, 238, 239]
Tunisia	EMRO	lower-middle-income	2	[240, 241]
Turkey	EURO	upper-middle-income	1	[220]
Vietnam	WPRO	lower-middle-income	2	[242, 243]
Zambia	AFRO	lower-middle-income	1	[69]
Zimbabwe	AFRO	lower-middle-income	3	[55, 56, 131]
Grand Total			175	

^*^Multi-country study

^**^American Samoa transitioned from a middle-income country to a high-income country in 2022 but was included in the review, given it met the criteria at the time of study publication

This review considers two broad categories of health conditions: non-communicable diseases (NCD) alone and mental health-associated conditions (mental health alone (MH) or combined NCD and mental health (NCD-MH)). Most studies (69%) focused on NCDs alone. Of those, cardiovascular diseases (including hypertension), cancers, chronic respiratory diseases, and diabetes were considered in 115 of the 175 (65.7%) studies. Articles focused on mental health alone or mental health and NCDs (MH + NCD-MH combined category) accounted for 31% of studies (Table 2). Mental Health disorders included depression, anxiety, schizophrenia, bipolar disorders, post-traumatic stress disorder, addictions, and obsessive–compulsive disorders; some papers included epilepsy and dementia as mental health disorders, and they were re-categorized under NCDs.

**Table 2:** Distribution of citations by health conditions

Health Condition	# Citation	% Health condition
**Non-communicable Diseases (NCD)**	**121**	69%*
Asthma and/or Chronic Obstructive Pulmonary Disease	4	3%
Cardiovascular Disease or CVD Risk Factors	15	12%
Hypertension	54	45%
Hypercholesteremia	3	2%
Prediabetes or Diabetes	63	52%
Kidney Disease or Risk Factors	3	2%
Cancer (Cervical, Breast, Colon)	13	11%
Neurological (Stroke, Epilepsy, Dementia)	6	5%
Mental Health (MH) and NCD–MH	**54**	**31%**
Total	**175**	**100%**

^*^Several papers described health conditions that fall into multiple categories and were counted in more than one category

When considering the role of the health workforce in delivering services for NCDs, the findings of the scoping review particularly aligned with factors related to multidisciplinary team service delivery, including the composition of teams and task-shifting or the skills mix of teams. Additional File 6: Appendix S7 presents the full data on health workers and their roles extracted from each study.

## Multidisciplinary team service delivery

One hundred twenty-five of 175 (71%) articles described multidisciplinary teams (MDTs) for managing NCDs in primary care. 67% of MDT-related articles focused on NCDs alone, including 35% for CVD/HTN, 34% for pre-diabetes/diabetes, and 9% for cancers. Over two-thirds of studies (69%) reported on MDTs for NCD-related services alone took place in upper-middle-income countries. 33% of MDT-related articles examined mental health or mental health and NCD combined service delivery programs. A higher proportion of programs that focused on mental health were delivered in lower-income and lower-middle-income settings (58%) compared to programs focused on NCD management (31%). To foster an understanding of the different roles and services that various health providers deliver, we disaggregate data based on the type of health workers.

### Health workers included in teams

**Fig. 3:**
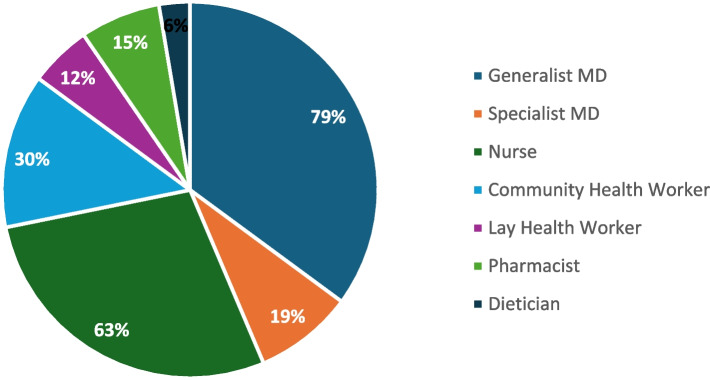
Health workers included in MDTs for NCD conditions

Among articles that described MDTs for the management of NCDs alone in all lower- and middle-income country settings, the teams included the following health workers: 79% reported on teams that included a generalist MD (which includes family physicians or general practitioners), 63% nurses, 30% community health workers (CHWs), 19% specialist physicians (including obstetricians, gynecologists, psychiatrists, pulmonologists, diabetes specialists or unspecified specialists), 12% lay health workers, 15% pharmacists or pharmacy technicians and 5% a dietician or nutritionist (Fig. 3). Other less commonly mentioned health workers included physical educators, physiotherapists, midwives, and dental personnel.

The distinction between CHWs and lay health workers was not consistent across articles. We opted to report any health worker explicitly described as a volunteer or a “lay worker”. However, we acknowledge that those described expressly as CHWs may also be volunteers and/or untrained health workers; alternatively, those described as “lay health workers” may have been provided with some training and/or a stipend. We however, recognize that both health worker categories constitute a diverse group of “non-professional” and community-anchored health workers.

**Fig. 4:**
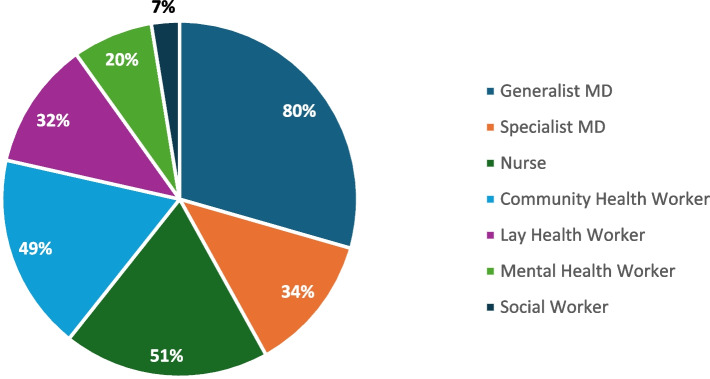
Health workers included in MDTs for mental health conditions and combined NCD and mental health programs

The various types of health workers included in teams were similar for articles that described MDTs for managing mental health and NCD and mental health combined programs. 80% reported a team that included a generalist MD, 51% nurses, 49% CHWs, 34% specialist physicians (psychiatrists), 32% lay health workers, 20% some form of mental health worker, and 7% included a social worker (Fig. 4). Other less commonly included health workers included auxiliary health workers and traditional faith healers.

### Service delivery tasks as part of a team

Where feasible, we extracted data on the tasks performed by the various health workers who were part of an MDT. Specifically, we looked at education and counselling, screening, diagnosis, treatment and referral [17]. Some papers outlined tasks for only some of the health workers described.

**Fig. 5:**
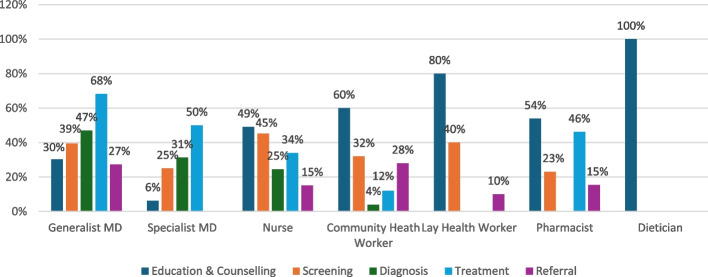
Services provided by MDT members for NCD conditions

When exploring the services delivered by health workers as part of MDTs for the management of NCDs alone (Fig. 5), we found that generalist physicians (which include family physicians and general practitioners) were most likely to be involved in diagnosis (47%) or treatment (68%). Similarly, specialist MDs were most likely engaged in diagnosis (31%) and treatment (50%). Nurses were most involved in education and counselling (49%) and screening (45%); however, compared to all our non-physician health workers, they were most likely to be involved with diagnosis (25%) and treatment (34%). Community health workers (CHWs) and lay health workers were most involved in education and counselling (60% and 80%, respectively) and screening (32% and 40%, respectively). Pharmacists were most involved in education and counselling (54%) and supporting treatment (46%). Dieticians were exclusively involved in education and counselling (100%).

**Fig. 6:**
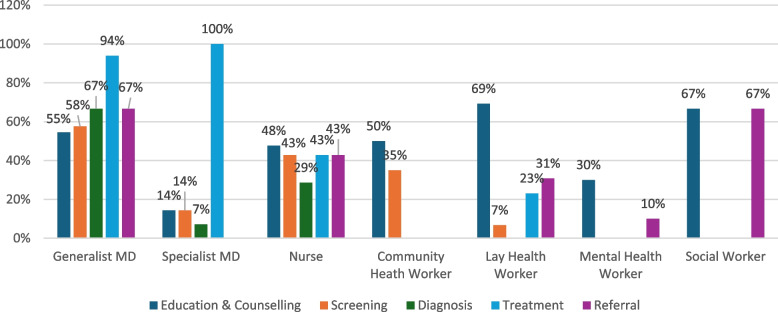
Services provided by MDT members for mental health conditions and combined NCD and mental health programs

When exploring the services delivered by health workers as part of MDTs for the management of mental health conditions alone or combined NCD and mental health programs (Fig. 6), we found again that generalist physicians (which include family physicians and general practitioners) were most likely to be involved in diagnosis (67%) or treatment (94%); however, in this grouping, they were more likely to be involved in referral (67%). Specialist MDs were most likely to be involved in treatment (100%). Diagnosis was only reported at 7% for specialist MDs. Nurses in these teams were equally involved in education, counselling, screening, treatment, and referral (43%). CHWs and lay health workers were most commonly involved in education and counselling (50% and 69%, respectively) but were also noted to be involved in supporting treatment adherence. We noticed that lay health workers were more involved in referrals for mental health-related programs (31%) than NCD-alone programs. Mental health workers were most engaged with education and counselling (30%), while social workers were equally involved in education, counselling and referrals (67%).

We also looked at team composition. We found that when a generalist MD was a part of the team (79% of teams), 51% of the time, they worked with a nurse, and 30% of the time, they worked with a CHW. 18% of teams included a generalist MD, nurse, and CHW. 7% of teams had a nurse with no associated generalist MD.

We also looked at examples of how MDTs were described in the literature. A study in Mali focused on training general practitioners to care for mental health disorders found that general practitioners were more confident in diagnosing and treating mental health disorders post-training[45]. It also highlighted how they worked alongside CHWs who collaborated with community leaders, such as traditional healers, which allowed them to improve community awareness of mental health and thus improve health-seeking behaviours[45]. An implementation study evaluated a mentoring and supervision program for primary mental health care services in Rwanda, where primary care nurses were responsible for diagnosing individuals presenting with mental health concerns, providing interventions including pharmacotherapy, psychoeducation, and linkages with social and community-based supports, and CHWs were responsible for case finding, promoting treatment adherence, psychoeducation, and stigma reduction[46]. The study found that the program led to increased service usage, enhanced delivery of mental health care by primary care nurses, and significant improvements in clinical symptoms and functional disability among users at supported health centers[46]. A randomized controlled trial in India evaluated the MANAS project, which aimed to promote mental health interventions led by lay health counsellors under the supervision of generalist physicians and mental health specialists[47]. The study assessed the effectiveness of collaborative stepped-care interventions and found that patients with common mental disorders in the intervention group were more likely to recover after six months compared to those in the control group.

A study in China evaluating a physician-pharmacist collaborative clinic for managing type 2 diabetes found significant improvements in medication compliance, quality of life, and fewer emergency visits among patients in the intervention clinic[48]. Similarly, in Mexico, a “shared medical appointment” model involving physicians, nurses, and CHWs was compared to appointments with physicians alone for diabetes management[49]. The MDT model made patients feel more comfortable, built trust, and improved support from peers and family. Physicians noted that delivering care as part of a team helped redistribute the burden of care, reallocating responsibilities and fostering teamwork.

Also, in China, healthcare reforms initiated since 2009 aimed to strengthen primary care by implementing measures to enhance the capacity, quality, and efficiency of primary care as well as rebuild the referral system to encourage residents to register with family doctor teams, which included family physicians, nurses, and public health physicians, who will act as gatekeepers of their medical care. A study comparing diabetic patients who received regular care from attached family doctor teams with those who had not registered to a team between 2015 and 2017 found that the family doctor team system promoted better continuity of care for diabetes management compared to usual care[50].

We found 16 articles conducted in Brazil. In Brazil, the Family Health Strategy, a core component of the publicly funded Unified Health System, involves a MDT model comprising a physician, a nurse, a nursing technician, and four CHWs on average at each basic PHC team. This team provides primary care to a geographically empanelled population, typically serving around 3,000 individuals[51]. One study aimed to assess the impact of CHWs on primary healthcare strategies targeting women's and children’s health, diabetes, and hypertension[52]. To do this, an independent variable termed "scope of home activities" was introduced to estimate the activities performed by CHWs, who were primarily responsible for home visits and active search efforts. The study found high levels of CHW activity were most strongly associated with hypertension management (OR = 6.88), followed by children’s health (OR = 6.56), women’s health (OR = 6.21), and diabetes care (OR = 5.64). These findings indicate that increased CHW involvement is linked to higher rates of health care and services, including active early detection and management of diabetes.

## Optimizing roles and skills mix of teams and task shifting

Optimizing roles within teams was a second central theme covered in 41 of 175 (23%) articles in this review. This refers to a process where specific clinical and administrative tasks are moved, where appropriate, to health workers with traditionally different scopes of practice, other training, or focused competency[53]. This can also be referred to as task shifting[54]. Among these 41 articles, task shifting was used for a wide range of NCDs and across a wide range of countries; 44% took place in lower middle-income countries, 7% in lower-income countries, and 49% in upper middle-income countries. Table 3 highlights the types of workers involved in such services optimization.

**Table 3:** Overview of task shifting between health workers by health conditions

Country income classification/Health condition	# of Citations	% of	Citation #
Lower Middle Income	**18**	**44%**	
MH*	5		[55–59]
NCD	13		
Nurse to CHW	1		[60]
Physician to CHW	1		[61]
Physician to Nurse	8		[62–69]
Physician to Team	1		[70]
Specialist to Nurse	1		[71]
Unspecified to CHW	1		[72]
Low Income	**3**	**7%**	
MH*	1		[46]
NCD	2		
Physician to CHW	1		[73]
Physician to Medical Assistant	1		[74]
Upper-Middle-Income	**20**	**49%**	
NCD	16		
HCW to LHW	2		[75, 76]
Nurse to LHW	2		[77, 78]
Physician to CHW	1		[79]
Physician to Non-Physician	2		[80, 81]
Physician to Nurse	4		[82–85]
Physician/Nurse to CHW	1		[86]
Team to Nurse	1		[87]
Unspecified to LHW	2		[88]
Unspecified	1		[89]
MH*	2		[90, 91]
NCD-MH*	2		
Total	**41**	**100%**	

^*^MH not specified by the health workers due to low numbers

Regarding the specific health workers, services were shifted from physicians to nurses in 13 of 41 articles[62–68, 83–85, 92, 94, 95]. One study examined the long-term (four years) glycemic outcome of a structured nurse-led intervention program for Type 2 Diabetes in rural South Africa, which found benefits in improving the HbA1c and was received well by patients and providers[84]. Another study assessed the implementation of a protocol-driven primary nurse-led care model for patients with Type 2 diabetes in rural and urban Cameroon and found that nurses delivered a management program for diabetes with positive medium-term outcomes in terms of glucose and blood pressure control at primary care levels[64].

Among the studies that discuss NCDs in lower-middle-income countries, three examined task-shifting related to cervical cancer screening from physicians to nurses [62, 69, 71]. In one of the studies, nurses were trained to offer Visual Inspection with Acetic Acid (VIA) screening to eligible women. This approach achieved a 95% agreement in diagnostic accuracy following the training. Additionally, it expands access to cervical cancer screening by integrating it into the broader preventive care services nurses provide [71].

Other studies describe shifting responsibilities from physicians and nurses to CHWs[60, 61, 72, 73, 79, 86]. For example, in hypertension management, CHWs can also accurately measure blood pressure and support care for hypertensive patients[73]. In a cluster-randomized controlled trial conducted in rural areas of Bangladesh, Pakistan, and Sri Lanka that evaluated a multi-component intervention for hypertension management, CHWs performed blood pressure monitoring and health education at home, only referring patients to physicians if necessary[96]. The study results showed that this intervention led to a more significant reduction in blood pressure compared to usual care.

Six of 41 (15%) articles explored shifting roles to lay health workers, all taking place in upper-middle-income countries[75–78, 88, 97]. Many studies highlighted how this group of health workers supports administrative processes. For instance, lay health workers in South Africa were integrated into nurse-led primary care centers to address increased demand and limited resources[77]. In the intervention clinics, the lay workers assumed various clinical and administrative roles, including measuring blood pressure, managing appointments, handling medical records, providing health education, assisting with medication prepacking, and appointment reminders and follow-ups. Overall, the support of lay health workers was welcomed and believed to support better adherence to appointments; however, there were concerns from some patients regarding confidentiality, as the lay health workers were from the same local community. Another study, a pragmatic cluster RCT, found no significant improvement in blood pressure control among patients in intervention clinics (i.e., those with lay health workers) compared to control clinics[78]. However, these lay health workers' presence enhanced clinic operations, including improving patient attendance and adherence to appointment schedules.

## Discussion

We conducted a scoping review of the literature to understand the role and organization of health workers in managing NCDs in primary care settings in LMICs.

We found that 72% of papers highlighted the role of MDTs in chronic disease management. When comparing programs for managing NCDs and mental health, it was interesting to observe that MDTs were more commonly involved in NCD-related care in upper middle-income countries and more commonly involved in mental health and combined NCD-MH programs in lower middle-income countries. One hypothesis could be that supporting MDTs is a relatively resource-intensive approach that may be difficult to replicate in lower-resource settings[98, 99]. Alternatively, there may have been a greater recent attention to MH and MH-NCD programs in lower-income countries due to a greater awareness of the need to address MH in the last two decades, and, as such, there has been a more intentional effort by governments and policymakers to financially prioritize support towards interventions to address this area[100, 101]. Lastly, the complexity and particular challenges of treating mental health issues in all settings may have prompted the involvement of a wider range of health workers[102].

Our findings align with a growing body of literature emphasizing the critical role of MDTs and task-shifting strategies in improving access and continuity of care in LMIC primary care settings. For example, Joshi et al. (2014) and Kane et al. (2017)emphasized the importance of task-shifting approaches and MDTs, particularly the redistribution of responsibilities from physicians to nurses or CHWs, in enhancing service coverage and continuity of care in resource-limited settings[103, 104]. Similarly, Ahmed et al. (2021)found that community-based delivery models involving CHWs improved access to conventional health services among marginalized groups, including rural populations, women, individuals from lower socio-economic backgrounds, and those with limited literacy[105]. Our review expands on these findings by comprehensively mapping the composition of primary care teams and the specific tasks delegated within those teams.

We found that teams most commonly include generalist physicians (which includes family physicians or general practitioners), followed by nurses and then community health workers. We also found that the type of non-physician health workers shifted depending on the health conditions addressed. For example, programs specifically looking at NCDs often included pharmacists and dieticians. In contrast, programs that included coverage for mental health disorders included mental health workers (which could consist of a variety of health professionals, including but not limited to psychologists, counsellors), and social workers. In these teams, regardless of health conditions treated, we found that physicians, either generalists or specialists, were mainly involved in diagnosis and treatment. While nurses played a broader role and significantly contributed to education, counselling, and screening for all health conditions. Nurses played a greater role in diagnosis and treatment in mental health-related programs. CHWs and lay health workers supported MDTs by providing education and screening. They were also noted to support services by raising awareness about programs, decreasing stigmatization about health conditions, and treatment adherence. Based on these findings, MDTs that address a broad spectrum of health conditions include, at a minimum, a generalist physician, nurse, CHW, pharmacist, dietician, mental health worker, and social worker. The exact composition of the team should be tailored to the specific health needs, socio-economic factors, and demographic characteristics of the community, as well as the size of the community being served[106, 107].Furthermore, depending on these factors, additional allied health professionals may be necessary to ensure that the team is equipped to address the unique health needs of the community in a comprehensive and sustainable manner[108].

One barrier to implementing MDTs commonly cited is the insufficient number of adequately trained health workers, especially specialists, in many LMICs [109–111]. Implementing policy to support training, deployment, and decentralization of qualified professionals for assessing and delivering care is important to achieving progress in NCD-related care, as for all primary care services[112–114]. Developing new, locally adapted models of care that recognize and potentially mitigate the constraints of limited resources is important. One way of achieving this is by implementing policies that supports redefining the roles, responsibilities, and scope of practice of existing health worker cadres and strategies to optimize their roles within teams[115, 116].

Relatedly, skill-mix innovations are an important aspect of MDTs that support the redistribution of tasks to optimize efficiency and the availability of existing cadres. This emerged as an important strategy for expanding the scope of NCD services, particularly in resource-constrained environments[117, 118]. Our findings align with a broader trend toward shifting clinical tasks from physicians to nurses and CHWs[119].This optimization of tasks through redistribution among health workers alleviates the burden on physicians, allowing them to concentrate on more complex tasks, and enhances service accessibility for patients[120, 121]. It also expands the scope of services provided by non-physician providers such as nurses and CHWs, and, in enabling a single health worker to potentially screen for multiple conditions such as cardiovascular disease, diabetes, cancers and mental health disorders, enables the integration of services and improves efficiency. Such integrated management of NCDs and mental health issues in a single facility or by a single provider or integrated team enhances the ability of the health system to address most of people’s common health needs in primary care.

### Limitations

While this scoping review provides a comprehensive examination of the role of health workers in chronic disease management in PHC settings within LMICs, certain limitations should be considered when interpreting the findings and their implications.

Although the review describes the common health workers in PHC-oriented programs in LMIC, based on a broad base of literature, this scoping review did not specifically explore how teams are formed, or how health workers collaborate within teams. Consequently, the review does not address factors related to team functioning.

Further research, including a systematic review or meta-analysis, is needed to understand the outcomes associated with different variations of team composition and task shifting within teams.

Also, some countries are more prominently represented than others, presenting potential biases in the findings. For instance, American Samoa, which does not neatly fit into the LMIC classification, stands out. While this diversity can enhance our understanding of different healthcare approaches, it may also obscure valuable information from underrepresented countries, potentially leading to an incomplete picture of the challenges and successes of NCD management within PHC settings across LMICs.

Additionally, although our literature search focused primarily on English-language studies, we searched a broad range of sources to facilitate inclusivity. Studies published in other languages were not reviewed but are available in Additional File 4: Appendix S3. Given non-English studies were not included in our review there is a possibility that valuable insights and perspectives were not captured, which could lead to gaps.

## Conclusion

This scoping review highlighted the integral role of multidisciplinary teams and the optimization of health workers' roles in managing non-communicable diseases in primary care settings. It is the first review to specifically look at the composition of teams and the tasks that health workers undertake. The core composition of teams is stable with generalist physicians, nurses, and community health workers, while the type of additional non-physician health workers is dependent on the most prevalent health conditions being addressed in the community served. Optimizing health workers' roles presents a valuable opportunity to redistribute responsibilities among health providers. Future research is needed to understand how primary care teams are formed, how individuals in teams best collaborate, and how to optimize roles within teams, to enhance chronic disease management in LMIC to improve efficiency and patient outcomes.

## Supplementary Information

Additional file 1.

Additional file 2.

Additional file 3.

Additional file 4.

Additional file 5.

Additional file 6.

## Data Availability

The datasets used and/or analyzed during the current study are available from the corresponding author on reasonable request.

## Abbreviations

AFRO: African Region
CHNs: Community Health Nurses
CHWs: Community Health Workers
CVD: Cardiovascular disease
DM: Diabetes Mellitus
EMRO: Eastern Mediterranean Region
EURO: European Region
HTN: Hypertension
LMICs: Low and middle-income countries
MDTs: Multidisciplinary teams
MH: Mental Health
NCD: Non-communicable Diseases
NCD-MH: Non-communicable Diseases and Mental Health Combined
PHC: Primary Health Care
SEARO: South-East Asian Region
VIA: Visual Inspection with Acetic Acid
WPRO: Western Pacific Region
