# Supplementary material for: Understanding the role and organization of health workers delivering non-communicable disease management in primary care in low- and middle-income countries: a scoping review
Source: BMC Prim Care. 2025 Nov 17;26:365. doi: 10.1186/s12875-025-03033-3 (PMC12625573; doi:10.1186/s12875-025-03033-3)
Supplement: Supplementary file 1 — Additional file 1. [file 12875_2025_3033_MOESM1_ESM.docx]

Additional file 1: Appendix M1

ELIGIBILITY CRITERIA

We applied inclusion criteria to identify studies that focused on models of care for NCDs within primary care settings in LMICs. We included studies that meet the following criteria: (1) address NCDs in clinical or non-clinical contexts; (2) specify the healthcare providers involved in the provision of care; (3) investigate, assess, or report on models of care integrating or delivering NCD services within primary care in LMICs; and (3) be published in the English language. Specifically, inclusion criteria for studies on models of NCD care within primary care or primary health care settings in LMICs consist of the following:

(a) Studies must have investigated or reported on service design, including (i) the delivery of services across diverse primary care platforms, *and/or* (ii) the establishment of a system to support first contact accessibility, *and/or* (iii) the establishment of care pathways to support continuity of care; **and/or**

(b) Studies must have examined or reported on organization and facility management, incorporating aspects such as (i) the involvement of primary care HCWs in the management of patients with NCDs, *and/or* (ii) specify which primary care providers are delivering care; *and/or* (iii) specify which services they are delivering, *and/or* (iv) the involvement of primary care HCWs in facility management, *and/or* (v) the engagement of MDTs in supporting integrated NCD management, *and/or* (vi) the involvement of primary care HCWs in supervising other healthcare providers; **and/or**

(c) Studies must have commented on the role of primary care HCWs in promoting community linkages and engagement, such as (i) collaboration with community members, *and/or* (ii) supporting proactive population outreach, *and/or* (iii) providing services for self-care and health literacy in primary care.

Our scoping review excluded study protocols, commentaries, studies conducted in high-income countries, editorials, case reports, and conference abstracts.

We identified non-English studies in the search, however, due to resource and time constraints, they are not included in this review.

Regarding the study designs incorporated in the review of models of care for NCD services within primary care in LMICs, we encompassed prospective and retrospective studies, case-control studies, and cross-sectional studies. Additionally, we consulted the reference lists of systematic reviews and scoping studies to identify additional relevant studies.
