# Supplementary material for: Understanding the role and organization of health workers delivering non-communicable disease management in primary care in low- and middle-income countries: a scoping review
Source: BMC Prim Care. 2025 Nov 17;26:365. doi: 10.1186/s12875-025-03033-3 (PMC12625573; doi:10.1186/s12875-025-03033-3)
Supplement: Supplementary file 2 — Additional file 2. [file 12875_2025_3033_MOESM2_ESM.docx]

**Appendix S1**

**WHO HCW NCD LMICS**

Database: Ovid MEDLINE(R) ALL <1946 to January 08, 2024>

Search Strategy:

--------------------------------------------------------------------------------

1 Health Personnel/ (66303)

2 ((health or healthcare or health care) adj (personnel or provider? or professional? or staff or work force? or workforce? or worker?)).tw,kw,kf. (214351)

3 Community Health Workers/ (6713)

4 ((community or community-based or local or village) adj3 (healer? or health worker? or health care worker? or healthcare worker?)).tw,kw,kf. (9384)

5 ((community or community-based or local or village) adj3 (healer? or health aide? or health care aide? or healthcare aide?)).tw,kw,kf. (425)

6 ((community or community-based or local or village) adj3 (FP or FPs or GP or GPs or NP or NPs)).tw,kw,kf. (1399)

7 health visitor?.tw,kw,kf. (2795)

8 Family Practice/ (67363)

9 General Practice/ (15748)

10 ((community or community-based or family* or general) adj practice?).tw,kw,kf. (59176)

11 family medicine.tw,kw,kf. (14227)

12 medical officer?.tw,kw,kf. (4106)

13 Nurses/ (46811)

14 Nurses, Community Health/ (1020)

15 Nurses, Public Health/ (552)

16 (nurse or nurses).tw,kw,kf. (316210)

17 (nursing adj (personnel or staff)).tw,kw,kf. (17591)

18 Physicians/ (103994)

19 Physicians, Family/ (17359)

20 Physicians, Primary Care/ (4431)

21 General Practitioners/ (10962)

22 (physician? or doctor? or general practitioner? or medical practitioner?).tw,kw,kf. (646373)

23 ((primary care or primary health care or primary healthcare) adj provider?).tw,kw,kf. (13929)

24 (PCP or PCPs).tw,kw,kf. (17840)

25 Primary Health Care/ and exp Workforce/ (3683)

26 acupuncturist?.tw,kw,kf. (842)

27 Counselors/ (686)

28 counsel?or?.tw,kw,kf. (11652)

29 Dentists/ (19832)

30 (dentist? or dental assistant? or dental hygienist? or dental technician? or dental therapist? or periodontist? or perio-dontist? or prosthodontist? or prostho-dontist?).tw,kw,kf. (48731)

31 Nutritionists/ (1812)

32 (dietician? or dietitian? or nutritionist?).tw,kw,kf. (13533)

33 Paramedics/ (129)

34 (paramedic? or para-medic? or ((paramedical or para-medical) adj2 personnel)).tw,kw,kf. (8087)

35 Pharmacists/ (22236)

36 (pharmacist? or pharmac* aide? or pharmac* technician?).tw,kw,kf. (45035)

37 Physical Therapists/ (3299)

38 (physical therapist? or physiotherapist? or physio-therapist? or physio or physios).tw,kw,kf. (22960)

39 Psychotherapists/ (208)

40 (psychotherapist? or psycho-therapist? or psychologist? or counse?lor?).tw,kw,kf. (34054)

41 Social Workers/ (1144)

42 (socialworker? or social worker?).tw,kw,kf. (12415)

43 Traditional Medicine Practitioners/ (36)

44 (alternative medic* practitioner? or complementary medic* practitioner? or CAM practitioner? or ethno-botanist? or ethnobotanist? or herbalist? or herbal medicine practitioner? or homeopath? or homeo-path? or ((indigenous? or traditional*) adj2 practitioner?) or medicine m#n or naturopath? or naturo-path? or shaman? or traditional healer?).tw,kw,kf. (10040)

45 (alternative medic* professional? or complementary medic* professional? or CAM professional? or herbal medicine professional? or ((indigenous? or traditional*) adj2 professional?)).tw,kw,kf. (440)

46 or/1-45 [**PRIMARY HEALTHCARE WORKFORCE**] (1402399)

47 Noncommunicable Diseases/ (3343)

48 ((noncommunicable or non-communicable or noninfectious* or non-infectious*) adj (condition? or disease? or disorder? or health or ill or illness* or sick or sickness*)).tw,kw,kf. (19807)

49 exp Chronic Disease/ (630535)

50 (chronic* adj (condition? or disease? or disorder? or health or ill or illness* or sick or sickness*)).tw,kw,kf. (151743)

51 ((multiple or multi-organ? or multiorgan? or multi-system? or multisystem?) adj3 (chronic* or condition? or disabl* or disabilit* or disease? or disorder? or illness* or sickness*)).tw,kw,kf. (72878)

52 ((chronic* or condition? or disabl* or disabilit* or disease? or disorder? or health or ill or illness* or sick or sickness* or medical*) adj3 complex*).tw,kw,kf. (88062)

53 exp Cardiovascular Diseases/ (2757264)

54 ((artery or arteries or arterial or atrial* or atrium* or cardiac* or cardio* or coronary or heart? or myocard* or ventricle*) adj3 (arrest* or aneurysm* or arrhythmia* or arrythmia* or disease* or disorder* or dysrhythmia* or dysrythmia* or dysfunction* or failure* or fibrillation* or ischemi* or infarction* or obstruct* or rupture* or syndrome*)).tw,kw,kf. (1231193)

55 (arterioscleros* or arterio-scleros* or atheroscleros* or athero-scleros*).tw,kw,kf. (154618)

56 (isch?emi* adj2 stroke?).tw,kw,kf. (83474)

57 (CVD and (artery or arteries or arterial or atrial* or atrium* or cardiac* or cardio* or coronary or heart? or myocard* or ventricle*)).tw,kw,kf. (44605)

58 (CHD and (artery or arteries or arterial or atrial* or atrium* or cardiac* or cardio* or coronary or heart* or myocard* or ventricle*)).tw,kw,kf. (29155)

59 exp Hypertension/ (321430)

60 Prehypertension/ (1174)

61 (hypertensi* or prehypertensi* or pre-hypertensi*).tw,kw,kf. (525787)

62 (blood pressure? adj3 (elevat* or high* or increas* or rais?)).tw,kw,kf. (72904)

63 (arter* adj2 pressure? adj3 (elevat* or high* or increas* or rais?)).tw,kw,kf. (19498)

64 (aortic* adj2 pressure? adj3 (elevat* or high* or increas* or rais?)).tw,kw,kf. (1017)

65 (systolic* adj2 pressure? adj3 (elevat* or high* or increas* or rais?)).tw,kw,kf. (13518)

66 (diastolic* adj2 pressure? adj3 (elevat* or high* or increas* or rais?)).tw,kw,kf. (7791)

67 exp Diabetes Mellitus/ (517872)

68 (diabet* or IDDM or MODY or NIDDM or T1DM or T1 DM or T2DM or T2 DM or Type 1 DM or Type 2 DM or gestational DM or adult-onset or early-onset DM or juvenile-onset DM or late-onset DM or noninsulin-dependent DM or insulin-dependent or prediabet* or pre-diabet*).tw,kw,kf. (821884)

69 exp Dyslipidemias/ (87776)

70 (dyslipid?emi* or dyslipoprotein?emi* or dyslipo-protein?emi* or hyperlip?emi* or hyper-lip?emi* or hyperlipid?emi* or hyper-lipid?emi* or lip?emi* or lipid?emi* or hypercholesteremi* or hypercholester?emi* or hypercholesterol?emi* or hyper-cholesterol?emi* or elevated cholesterol* or high* cholesterol* or hypertriglycerid?emi* or hyper-triglycerid?emi* or elevated triglyceride* or high* triglyceride* or hyperlipoprotein?emi* or hyper-lipoprotein?emi*).tw,kw,kf. (151423)

71 exp Kidney Disease/ (581160)

72 ((kidney? or renal*) adj3 (disease? or disorder? or dysfunction* or patholog*)).tw,kw,kf. (236265)

73 (CKD or CKDs).tw,kw,kf. (45742)

74 exp Mental Disorders/ (1457951)

75 ((affective or behavio?r$2 or mental$2 or mood? or psychiatric* or psycholog* or psychotic) adj3 (condition? or disease? or disorder? or ill or illness* or malad* or sick or sickness*)).tw,kw,kf. (303027)

76 ((bi-polar or bipolar or manic) adj2 (disorder* or psychos#s)).tw,kw,kf. (42809)

77 (mania? or manic or manic-depressi*).tw,kw,kf. (20761)

78 (schizophreni* or schizo-affect* or schizoaffect* or schizophreniform* or schizo-phreniform*).tw,kw,kf. (147636)

79 (dementia adj (praecox or precox)).tw,kw,kf. (586)

80 (share$1 adj1 paranoid disorder*).tw,kw,kf. (22)

81 (share$1 adj1 psychotic disorder*).tw,kw,kf. (72)

82 ("folie a deux" or "folie a trois" or hebephreni*).tw,kw,kf. (595)

83 ((deficit or negative or positive) adj (symptom? or syndrome?)).tw,kw,kf. (19179)

84 ((brief psychotic adj2 disorder*) or (first psychotic episode* or 1st psychotic episode* or first episode psychos* or 1st episode psychos*)).tw,kw,kf. (5186)

85 Depression/ (154509)

86 (depress* or dysthym* or blues or melanchol* or MDD).tw,kw,kf. (586969)

87 exp Anxiety/ (115035)

88 (angst or anxiet* or anxious* or catastrophi* or hypervigilan* or hyper-vigilan* or nervous*).tw,kw,kf. (679229)

89 ((incessan* or obsessive* or persistan* or intense*) adj3 fear*).tw,kw,kf. (585)

90 (agoraphobi* or claustrophobi*).tw,kw,kf. (4324)

91 (neuro-circulatory asthenia* or neurocirculatory asthenia* or cardiac neuros#s or effort syndrome* or hyper-kinetic heart syndrome* or hyperkinetic heart syndrome*).tw,kw,kf. (677)

92 (neurotic disorder* or neuros#s or psychoneuros#s or psycho-neuros#s).tw,kw,kf. (12275)

93 (obsessive-compulsive or anankastic personalit* or compulsive personalit* or obsessive personalit* or hoarding or psychastheni*).tw,kw,kf. (23525)

94 (panic disorder* or (panic adj2 attack*)).tw,kw,kf. (12378)

95 (phobia or phobias or phobic).tw,kw,kf. (13251)

96 exp Neoplasms/ (3919208)

97 (neoplas* or cancer* or tumour* or tumor* or carcinoma* or malignan* or metasta* or oncolog*).tw,kw,kf. (4216982)

98 (adenoma? or adenocarcinoma? or adeno-carcinoma? or blastoma? or carcinosarcoma? or carcino-sarcoma? or hepatoblastoma? or hepato-blastoma? or leukemia? or leukaemia? or lymphoma? or melanoma? or mesenchymoma? or mesothelioma? or sarcoma? or thymoma?).tw,kw,kf. (978617)

99 or/47-98 [**CHRONIC CONDITIONS/NON-COMMUNICABLE DISEASES**] (11719528)

100 46 and 99 [**HEALTHCARE WORKFORCE - CHRONIC CONDITIONS/NON-COMMUNICABLE DISEASES**] (424950)

101 Delivery of Health Care/ (120431)

102 Delivery of Health Care, Integrated/ (14392)

103 Intersectoral Collaboration/ (2583)

104 Continuity of Patient Care/ (20775)

105 ((allied or integrat* or collaborat* or comprehensive* or consolidat* or continual* or continuous* or continuit* or continuum? or co-ordinat* or coordinat* or inter-disciplin* or interdisciplin* or inter-professional* or interprofessional* or inter-sectoral* or intersectoral* or multi-disciplin* or multidisciplin* or multi-professional* or multiprofessional*) adj5 (care or deliver* or framework? or health care or healthcare or model? or service? or system?)).tw,kw,kf. (331169)

106 Models, Organizational/ (19557)

107 or/101-106 [**INTEGRATED CARE**] (472957)

108 100 and 107 [**HEALTHCARE WORKFORCE - NCD - INTEGRATION**] (34564)

109 Primary Health Care/ (92909)

110 (primary care or primary health care or primary healthcare).tw,kw,kf. (181058)

111 ((first or initial* or original* or preliminary or primary or start*) adj3 (access$2 or appointment? or contact? or encounter? or meeting? or POC or visit?)).tw,kw,kf. (62601)

112 or/109-111 [**PRIMARY HEALTH CARE**] (255436)

113 108 and 112 [**HEALTHCARE WORKFORCE - NCD - INTEGRATION - PRIMARY CARE**] (9361)

114 (afghan* or africa* or albania* or algeria* or angola* or antigua* or barbuda* or argentin* or armenia* or aruba* or azerbaijan* or bahrain* or bangladesh* or bengal* or bangal* or barbados* or barbadian* or bajan or bajans or belarus* or belorus* or byelarus* or byelorus* or belize* or benin* or dahomey or bhutan* or bolivia* or bosnia* or herzegovin* or botswan* or batswan* or bechuanaland* or brazil* or brasil* or bulgaria* or burkina* or burkinese* or upper volta* or burundi* or urundi* or cabo verde* or cape verde* or cambodia* or kampuchea* or khmer* or cameroon* or cameroun* or ubangi shari* or chad* or chile* or china* or chinese or colombia* or comoro* or comore* or comorian* or mayotte* or congo* or zaire* or costa rica* or "cote d'ivoir*" or "cote d' ivoir*" or cote divoir* or cote d ivoir* or ivory coast* or ivorian* or croatia* or cuba or cuban or cubans or "cuba's" or cyprus* or cypriot* or czech* or djibouti* or french somaliland* or dominica* or ecuador* or egypt* or united arab republic* or el salvador* or salvadoran* or guinea* or equatoguinea* or eritrea* or estonia* or eswatini* or swaziland* or swazi* or swati* or ethiopia* or fiji* or gabon* or gabonese* or gabonaise* or gambia* or ((georgia or georgian or georgians) not (atlanta or california or florida)) or ghana* or gibraltar* or greece* or greek* or grecian* or grenada* or grenadian* or guam* or guatemala* or guyana* or guiana* or guyanese* or haiti* or hispaniola* or hondura* or hungary* or hungarian* or india* or indonesia* or iran* or iraq* or isle of man* or jamaica* or jordan* or kazakh* or kenya* or karabati* or korea* or kosovo* or kosova* or kyrgyz* or kirgiz* or kirghiz* or laos or lao or laotian* or latvia* or lebanon* or lebanese* or lesotho* or lesothan* or lesothonian* or basutoland* or mosotho* or basotho* or liberia* or libya* or jamahiriya* or lithuania* or macedonia* or madagasca* or malagasy* or malawi* or nyasaland* or malaysia* or malay* federation or maldives* or maldivian* or indian ocean or mali or malian* or "mali's" or malta or maltese* or "malta's" or micronesia* or marshallese* or kiribati* or marshall island* or nauru or nauran or nauruans or "naurian's" or mariana or marianas or palau or paluan* or tuvalu* or mauritania* or mauritan* or mauritius* or mexico* or mexican* or moldova* or moldovia* or mongol* or montenegr* or morocco* or moroccan* or ifni or mozambique* or mozambican* or myanmar* or burma* or burmese or namibia* or nepal* or new caledonia* or netherlands antill* or nicaragua* or niger* or oman or omani or omanis or "oman's" or pakistan* or palestin* or gaza* or west bank* or panama* or paraguay* or peru or peruvian* or "peru's" or philippine* or philipine* or phillipine* or phillippine* or filipino* or filipina* or poland* or polish or pole or poles or portugal* or portuguese or puerto ric* or romania* or russia* or ussr* or soviet* or rwanda* or rwandese or ruanda* or ruandese or samoa* or navigator island* or pacific island* or polynesia* or "sao tome and principe*" or sao tomean* or santomean* or saudi arabia* or saudi or saudis or senegal* or serbia* or seychell* or sierra leone* or slovak* or sloven* or melanesia* or solomon island* or norfolk island* or somali* or sri lanka* or ceylon* or "saint kitts and nevis*" or "st kitts and nevis*" or kittian* or nevisian* or saint lucia* or st lucia* or saint vincent* or st vincent* or vincentian* or grenadine* or sudan* or surinam* or syria* or tajik* or tadjik* or tadzhik* or tanzania* or tanganyika* or thai* or timor leste* or east timor* or timorese* or togo or togoles* or "togo's" or tonga* or trinidad* or tobago* or tunisia* or turkiy* or turkey* or turk or turks or turkish or turkmen* or uganda* or ukrain* or uruguay* or uzbek* or vanuatu* or new hebrides* or venezuela* or vietnam* or viet nam* or yemen* or yugoslav* or zambia* or zimbabwe* or rhodesia* or arab* countr* or middle east* or global south or sahara* or subsahara* or magreb* or maghrib* or west indies* or caribbean* or central america* or latin america* or south america* or central asia* or north asia* or northern asia* or southeastern asia* or south eastern asia* or southeast asia* or south east asia* or west asia* or western asia* or east europe* or eastern europe* or developing countr* or developing nation* or developing population* or developing world or less developed countr* or less developed nation* or less developed world or lesser developed countr* or lesser developed nation* or lesser developed world or under developed countr* or under developed nation* or under developed world or underdeveloped countr* or underdeveloped nation* or underdeveloped world or middle income countr* or middle income nation* or middle income population* or low income countr* or low income nation* or low income population* or lower income countr* or lower income nation* or lower income population* or underserved countr* or underserved nation* or underserved population* or under served population* or under served nation* or under served population* or deprived countr* or deprived population* or high burden countr* or high burden nation* or countdown countr* or countdown nation* or poor countr* or poor nation* or poor population* or poor world or poorer countr* or poorer nation* or poorer population* or poorer world or developing econom* or less developed econom* or underdeveloped econom* or under developed econom* or middle income econom* or low income econom* or lower income econom* or low gdp or low gnp or low gross domestic or low gross national or lower gdp or lower gnp or lower gross domestic or lower gross national or lmic or lmics or third world or lami countr* or transitional countr* or emerging econom* or emerging nation*).ti,ab,hw,kw,kf. [**COCHRANE EPOC LMIC FILTER - 2022**] (3284328)

115 113 and 114 [**HEALTHCARE WORKFORCE - NCD - INTEGRATION - PRIMARY CARE - LMICS**] (1609)

116 exp Animals/ not Humans/ (5184695)

117 115 not 116 [**ANIMAL-ONLY REMOVED**] (1609)

118 (editorial or news or newspaper article).pt. (916819)

119 117 not 118 [**OPINION PIECES REMOVED**] (1604)

120 Case Reports.pt. or (case report? or case study or case studies).ti,kw,kf. (2479659)

121 119 not 120 [**CASE REPORTS REMOVED**] (1575)

***************************
