# Supplementary material for: Understanding the role and organization of health workers delivering non-communicable disease management in primary care in low- and middle-income countries: a scoping review
Source: BMC Prim Care. 2025 Nov 17;26:365. doi: 10.1186/s12875-025-03033-3 (PMC12625573; doi:10.1186/s12875-025-03033-3)
Supplement: Supplementary file 4 — Additional file 4. [file 12875_2025_3033_MOESM4_ESM.docx]

**Appendix S3**

**Articles not in English**

1. Adler MS, Gallian DMC. Escola médica e Sistema Único de Saúde (SUS): criação do curso de medicina da Universidade Federal de São Carlos, SP, Brasil (UFSCar) sob perspectiva de docentes e estudantes. Interface (Botucatu). 2018;22(64):237–49. doi:10.1590/1807-57622015.0455. [Portuguese]
2. Affonso MVG, Pereira CEA, Silva WB, Silva MVS. O papel dos Determinantes Sociais da Saúde e da Atenção Primária à Saúde no controle da COVID-19 em Belém, Pará. Physis. 2021;31(2):e310207. doi:10.1590/S0103-73312021310207. [Portuguese]
3. Afkar M, Rezanejad Asl P, Mahdavi Hezaveh A, Akrami F, Riazi-Isfahani S, Peykari N, et al. The effect of Covid-19 pandemic on non-communicable disease prevention and management services in the primary health care system of Iran. Sci J Kurdistan Univ Med Sci. 2021;26(5):33–49. doi:10.52547/sjku.26.5.33. [Persian]
4. Alcayaga C, Perez JC, Bustamante C, Campos S, Lange I, Zuniga F. Plan piloto del sistema de comunicación y seguimiento móvil en salud para personas con diabetes. Rev Panam Salud Publica. 2014;35(5–6):458–64. [Spanish]
5. Alfena MD. Uso de psicotrópicos na Atenção Primária [dissertação]. Rio de Janeiro: Escola Nacional de Saúde Pública Sérgio Arouca, Fundação Oswaldo Cruz; 2015. [Portuguese]
6. Almeida PF, Fausto MCR, Giovanella L. Fortalecimento da atenção primária à saúde: estratégia para potencializar a coordenação dos cuidados. Rev Panam Salud Publica. 2011;29(2):84–95. [Portuguese]
7. Alvarenga EC, Oliveira PTR, Pinheiro HHC, Carneiro VCCB. Condições de trabalho de equipes de saúde da família do Pará. Rev Nufen Phenom Interd. 2018;10(1):58–72. [Portuguese]
8. Alvarenga MRM. Avaliação da capacidade funcional, do estado de saúde e da rede de suporte social do idoso atendido na Atenção Básica. BR; 2008. [Portuguese]
9. Alves CMP, Serralha CA. A Assistência Psicológica a Crianças em Unidades Básicas de Saúde. Estudos e pesquisas em psicologia. 2019;18(3):912–31. doi:10.12957/epp.2018.40460. [Portuguese]
10. Costa-Alves PR, Alexandre C da S, Macedo LC, Ladchumananandasivam FR, Oliveira ER, Sousa ESS. Coordenação de cuidados primários para o paciente com doença renal crônica em diálise: revisitando papéis. Revista de APS. 2022;24(Supl 1):200-218. [Portuguese]
11. Alves RB, Bruning N de O, Kohler KC. “O Equilibrista”: Atuação do Psicólogo no NASF no Vale do Itajaí. Psicologia, ciência e profissão. 2019;39():e186600-e186600. doi:10.1590/1982-3703003186600. [Portuguese]
12. Amaral RP, Tesser CD, Müller P. Benefícios dos grupos no manejo da hipertensão arterial sistêmica: percepções de pacientes e médicos. Rev Bras Med Fam Comunidade. 2013;8(28):196–202. doi:10.5712/rbmfc8(28)762. [Portuguese]
13. Amoras JAB. Ações de saúde da mulher desenvolvidas pela equipe de enfermagem da estratégia saúde da família no Mato Grosso do Sul [dissertação]. Campo Grande (MS): Universidade Federal de Mato Grosso do Sul; 2017:101. [Portuguese]
14. Andrade AB de, Bosi MLM. Qualidade do cuidado em dois centros de atenção psicossocial sob o olhar de usuários. Saúde e sociedade. 2015;24(3):887–900. doi:10.1590/S0104-12902015131949. [Portuguese]
15. Andrade AGM, Neves R da F, Carvalho R de CP, Dias EC, Lima MAG de. (In)Visibilidade do usuário-trabalhador na interação com profissionais de saúde no contexto de uma Unidade de Saúde da Família. Interface (Botucatu, Brazil). 2021;25: e200700-e200700. doi:10.1590/interface.200700. [Portuguese]
16. Andrade FAC. Acolhimento: ferramenta para a sistematização da assistência de enfermagem na atenção primária [dissertação]. Ribeirão Preto: Universidade de São Paulo, Escola de Enfermagem de Ribeirão Preto; 2020 [citado 2025-05-01]. doi:10.11606/D.22.2020.tde-06072020-140311. [Portuguese]
17. Arce VAR, Sousa MF de, Lima M da G. A práxis da saúde mental no âmbito da estratégia saúde da família : contribuições para a construção de um cuidado integrado. 2011;21(2):541-560. doi:10.1590/S0103-73312011000200011. [Portuguese]
18. Angelini CR, Caccia-Bava MCG. “A gente sente que precisa e pode...”: os desafios para a inclusão da saúde mental na Atenção Básica. Rev Bras Med Fam Comunidade. 2015;10(36):1 doi:10.5712/rbmfc10(36)1033. [Portuguese]
19. Antunes B. O apoio matricial em saúde mental na Estratégia da Saúde da Família : concepção da equipe de apoiadores. 2015;():96-96. [Portuguese]
20. Araújo EMD, Galimbertti PA. A colaboração interprofissional na estratégia saúde da família. Psicologia & Sociedade. 2013;25(2):461–8. doi:10.1590/S0102-71822013000200023. [Portuguese]
21. Araújo MLA, Medeiros AP, Zuculin S, Souza EG, Barros PF, Boaventura T, et al. Educação em saúde – estratégia de cuidado integral e multiprofissional para gestantes. Revista da ABENO. 2013;11(2):8–13. [Portuguese]
22. Arona E da C. Implantação do matriciamento nos serviços de saúde de Capivari. Saúde e sociedade. 2009;18(suppl 1):26–36. doi:10.1590/S0104-12902009000500005. [Portuguese]
23. Contatore OA. Cuidado, acupuntura e atenção primária à saúde: conceitos em construção e correlação [tese de doutorado]. Campinas (SP): Universidade Estadual de Campinas, Faculdade de Ciências Médicas; 2020. [Portuguese]
24. Ramos MAA. Política de saúde prisional brasileira: uma análise a partir da reforma sanitária [dissertação]. Rio de Janeiro: Escola Nacional de Saúde Pública Sergio Arouca, Fundação Oswaldo Cruz; 2014. 127 p. [Spanish]
25. Barbosa Júnior DA. A judicialização da política pública de assistência farmacêutica: análise a partir de uma abordagem deliberativa [tese]. Sao Paolo: Faculdade de Salude Publica da USP; 2023. [Portuguese]
26. Barbosa M de C, Mattos EBT, Mendes R. Capacitating community health workers to detect dementia at the mild stage/Capacitacao de agentes comunitarios de saude para a deteccao da demencia na fase leve/Capacitación de agentes comunitarios de salud para detección de las demencias en la fase leve. Revista enfermagem UERJ. 2019;27 ():e42244-:e42244. doi:10.12957/reuerj.2019.42244. [Portuguese]
27. Barbosa TL. Desafios na construção de uma Rede de Atenção Psicossocial no município de Manaus (AM): discursos e práticas de gestores e profissionais [tese de doutorado]. Rio de Janeiro: Universidade do Estado do Rio de Janeiro, Instituto de Medicina Social; 2019. [Portuguese]
28. Barrioso PDC. Cuidados paliativos e atenção primária à saúde: proposição de um rol de ações de enfermagem [dissertacao]. São Paulo: Escola de Enfermagem da Universidade de São Paulo; 2017. [Portuguese]
29. Bellenzani R, Mendes R de F. Entre o empenho, o acolhimento e a impotência: dilemas de agentes comunitárias de saúde na produção do cuidado e da humanização. Cadernos de Terapia Ocupacional da UFSCar. 2012;20(2):239–53. [Portuguese]
30. Ben Mansour N, Sassi Mahfoudh A, Ben Romdhane H. Management of diabetics. Comparative study of two contrasting health structures. La Tunisie medicale. 2021;99(1):129–38. [French]
31. Bezerra IC, Jorge MSB, Gondim APS, Lima LL de, Vasconcelos MGF. “Fui lá no posto e o doutor me mandou foi pra cá”: processo de medicamentalização e (des)caminhos para o cuidado em saúde mental na Atenção Primária. Interface (Botucatu, Brazil). 2014;18(48):61–74. [Portuguese]
32. Böing E, Crepaldi MA. O psicólogo na atenção básica: uma incursão pelas políticas públicas de saúde brasileiras. Psicologia: Ciência e Profissão. 2010;30(3):634–49. [Portuguese]
33. Bottari CM de S, Vasconcellos MM, Mendonça MHM de. Câncer cérvico-uterino como condição marcadora: Uma proposta de avaliação da atenção básica. Cadernos de saúde pública. 2008;24(1):S111–22. [Portuguese]
34. **Bouza Plasencia G, Villoch Bonet R, Plasencia Domínguez O, Sosa Tejeda I.** Calidad de la atención al anciano en dos policlínicos del municipio de Santiago de Cuba. MEDISAN*.* 2021;25(1):51–65. [Spanish]
35. Braga JA da C, Bordoni MZB, Brito E de, Mendes M dos S, Barros IFO de, Leon EB de. Potencialidades e fragilidades institucionais no cuidado ao idoso com hipertensão. Revista de Enfermagem da UFSM. 2022;12():e30-. doi:10.5902/2179769267615. [Portuguese]
36. Brito L de AR, Abreu M de. Identificação de competências necessárias aos médicos da Atenção Primária à Saúde para a entrega de cuidados paliativos ao paciente com insuficiência cardíaca. Revista brasileira de medicina de família e comunidade. 2022;17(44):3194-. doi:10.5712/rbmfc17(44)3194. [Portuguese]
37. Burlandy L, Mazalotti Teixeira MR, Cerqueira Castro LM, Cunha Cruz MC, Bocca Santos CR, de Souza SR, et al. Models of care for individuals with obesity in primary healthcare in the state of Rio de Janeiro, Brazil. Cadernos de saúde pública. 2020;36(3):e00093419-. doi:10.1590/0102-311x00093419. [Portuguese]
38. **Cabo DJV.** Homeopatia na Estratégia Saúde da Família: apoio matricial e visão sistêmica cartografando a integralidade do cuidado [dissertação]. Rio de Janeiro: Escola Nacional de Saúde Pública Sérgio Arouca; 2013. 77 f. [Portuguese]
39. Cabral IE, Motta IS da, Pimentel TGP, Corrêa MP de O, Arrué AM, Neves ET. Demandas de crianças com necessidades especiais de saúde na atenção primária da cidade do Rio de Janeiro. Ciência, cuidado e saúde. 2020;19. [Portuguese]
40. **Caçapava JR.** O acolhimento e a produção do cuidado em saúde mental na atenção básica: uma cartografia do trabalho em equipe [dissertação]. São Paulo: Escola de Enfermagem, Universidade de São Paulo; 2008. [Portuguese]
41. Calheiros MNT da R, Wyszomirska RM de AF, Omena KVM de, Calheiros D dos S. A educação permanente no âmbito da saúde mental e o médico atuante na atenção primária. Revista de APS. 2022;25(Supl 1):29-40. [Portuguese]
42. Camargo Jr KR de, Campos EMS, Bustamante-Teixeira MT, Mascarenhas MTM, Mauad NM, Franco TB, et al. Avaliação da atenção básica pela ótica político-institucional e da organização da atenção com ênfase na integralidade. Cadernos de saúde pública. 2008;24(suppl 1):s58–68. [Portuguese]
43. Camelo SHH, Angerami ELS. Formação de recursos humanos para a estratégia de saúde da família. Ciência, cuidado e saúde. 2008;7(1):45-52. [Portuguese]
44. Campos DB, Bezerra IC, Jorge MSB. PRODUÇÃO DO CUIDADO EM SAÚDE MENTAL: PRÁTICAS TERRITORIAIS NA REDE PSICOSSOCIAL. Trabalho, Educação e Saúde. 2020;18(1):1-. doi:10.1590/1981-7746-sol00231. [Portuguese]
45. Campos RO, Gama CA, Ferrer AL, Santos DVD dos, Stefanello S, Trapé TL, et al. Saúde mental na atenção primária à saúde: estudo avaliativo em uma grande cidade brasileira. Ciência & saude coletiva. 2011;16(12):4643–52. doi:10.1590/S1413-81232011001300013. [Portuguese]
46. Cardoso JR. Doenças crônicas não transmissíveis no contexto da Estratégia Saúde da Família: fabricando formas de gestão do cuidado [dissertação]. Rio de Janeiro (RJ): Escola Nacional de Saúde Pública Sergio Arouca, Fundação Oswaldo Cruz; 2014. [Portuguese]
47. Teixeira Carneiro C, Rocha Bezerra MA, Cardoso Rocha R, De Assis Brito M, Kanashiro Meneghetti F. Fluxos de atendimento às mulheres em situação de violência na atenção primária à saúde. Revista Ciência Plural. 2022;8(3):1–20. doi:10.21680/2446-7286.2022v8n3. [Portuguese]
48. Carriello LSSC. Tecendo teias: a construção de uma ferramenta tecnológica de notificação das internações sensíveis à atenção básica [dissertação]. Niterói (RJ): Universidade Federal Fluminense, Escola de Enfermagem Aurora de Afonso Costa; 2021. [Portuguese]
49. Carvalho BG. Coordenação de unidade da atenção básica no SUS: trabalho, interação e conflitos [tese]. São Paulo: Escola de Enfermagem, Universidade de São Paulo; 2012. [Portuguese]
50. Carvalho FW. Educação alimentar e nutricional na atenção primária à saúde na perspectiva do profissional não nutricionista [dissertação]. São Paulo: Universidade de São Paulo, Faculdade de Odontologia, Faculdade de Saúde Pública e Escola de Enfermagem; 2015. [Portuguese]
51. Castro SM, Rudolph CI, Aguilar JE. Psychiatric consulting in primary care. Medwave. 2013 Mar;13(3):e5646. doi: 10.5867/medwave.2013.03.5646. [Spanish]
52. Cavalcante SAM, Silva FB, Marques CAV, Figueiredo EN, Gutiérrez MGR. Ações do enfermeiro no rastreamento e diagnóstico do câncer de mama no Brasil*.* Rev Bras Cancerol*.* 2013;59(3):459–66. [Portuguese]
53. Cela M, Oliveira IF. O psicólogo no Núcleo de Apoio à Saúde da Família: articulação de saberes e ações. Estud Psicol (Natal). 2015;20(1):31–9. doi:10.5935/1678-4669.20150005. [Portuguese]
54. Cerqueira AVS, Pinheiro APT. Guia orientador para o enfrentamento da pandemia: relato do uso em um município da Bahia*.* Rev Baiana Saúde Pública*. 2021;45*(Esp 3):65–70. doi:10.22278/2318-2660.2021.v45.nEspecial_3.a3543. [Portuguese]
55. Cezar PK, Rodrigues PM, Arpini DM. A Psicologia na Estratégia de Saúde da Família: vivências da residência multiprofissional*.* Psicol Cienc Prof*. 2015;*35(1):211–24. doi:10.1590/1982–3703000012014. [Portuguese]
56. Chaves AFL, Pereira UL, da Silva AM, Caldini LN, Lima LC, de Vasconcelos HCA. Percepções de enfermeiros da atenção primária à saúde sobre o cuidado a pacientes oncológicos. Enferm Foco. 2020;11(2):91–7. doi:10.21675/2357-707X.2020.v11.n2.2880. [Portuguese]
57. Chaves LA, Jorge AO, Cherchiglia ML, Reis IA, Santos MAC, Santos AF, Machado ATGM, Andrade EIG. Integração da atenção básica à rede assistencial: análise de componentes da avaliação externa do PMAQ-AB. Cad Saúde Pública. 2018;34(2):e00201515. doi:10.1590/0102-311X00201515. [Portuguese]
58. Chazan LF, Fortes S, Camargo KR Jr, Freitas GC. O apoio matricial na Atenção Primária em Saúde no município do Rio de Janeiro: uma percepção dos matriciadores com foco na Saúde Mental. Physis Rev Saúde Coletiva. 2019;29(2):e290212. [Portuguese]
59. Cintra TS, Doricci GC, Guanaes-Lorenzi C. Dinâmicas relacionais de equipes na Estratégia de Saúde da Família. Rev SPAGESP. 2019;20(1):24–38. [Portuguese]
60. Clemente A, Matos DR, Grejanin DKM, Santos HE, Quevedo MP, Massa PA. Residência multiprofissional em saúde da família e a formação de psicólogos para a atuação na atenção básica. Saúde Soc. 2008;17(1):176–84. [Portuguese]
61. Conill EM. Ensaio histórico-conceitual sobre a Atenção Primária à Saúde: desafios para a organização de serviços básicos e da Estratégia Saúde da Família em centros urbanos no Brasil. Cad Saúde Pública. 2008;24(Suppl 1):S7–27. [Portuguese]
62. Cordeiro GFT, Santos TM dos, Ferreira RG dos S, Guljor APF, Peters AA, Peres MA de A. Protocolos para atendimento de saúde mental na atenção primária: subsídios para transformação da assistência. Cogitare Enferm. 2022;27:e82680. doi:10.5380/ce.v27i0.82680. [Portuguese]
63. Correia VR, Barros S, Colvero LA. Saúde mental na atenção básica: prática da equipe de saúde da família. Rev Esc Enferm USP. 2011;45(6):1501–6. doi:10.1590/S0080-62342011000600032. [Portuguese]
64. Costa EM, Rabelo ARM, Lima JG. Avaliação do papel do farmacêutico nas ações de promoção da saúde e prevenção de agravos na atenção primária. Rev Ciênc Farm Básica Apl. 2014;35(1):81–8. [Portuguese]
65. Costa GMC, Celino SM, Coura AS. Saúde-doença mental na atenção primária: uma prática assistencial em construção. Rev APS. 2012;15(4):479–85. [Portuguese]
66. Costa JMBS, Silva MRF, Carvalho EF. Avaliação da implantação da atenção à hipertensão arterial pelas equipes de Saúde da Família do município do Recife (PE, Brasil). Cien Saude Colet. 2011;16(2):623–33. doi:10.1590/S1413-81232011000200026. [Portuguese]
67. Costa MFBNA. Atenção integral à saúde do idoso na atenção primária: os sistemas brasileiro e espanhol [tese]. São Paulo (SP): Escola de Enfermagem, Universidade de São Paulo; 2009. [Portuguese]
68. Cotrim TM. O trabalho do enfermeiro no atendimento às gestantes: ações básicas, problemas comuns e a sistematização da assistência na consulta pré-natal [dissertação]. Ribeirão Preto (SP): Escola de Enfermagem de Ribeirão Preto, Universidade de São Paulo; 2020. [Portuguese]
69. Cueto-Manzano AM. El médico primario y la enfermedad renal crónica. Rev Invest Clin*.* 2008;60(6):517–26. [Spanish]
70. Cunha EM. Vínculo longitudinal na Atenção Primária: avaliando os modelos assistenciais do SUS [tese]. Rio de Janeiro (RJ): Escola Nacional de Saúde Pública Sergio Arouca, Fundação Oswaldo Cruz; 2009. [Portuguese]
71. D’Angelo SB, Palladino AC, Gómez JF. La satisfacción de la atención: una forma de integrar la comunidad a los servicios de salud. Rev Fac Med UNNE. 2016;36(2):12–21. [Spanish]
72. Dantas NF, Passos ICF. Apoio matricial em saúde mental no SUS de Belo Horizonte: perspectiva dos trabalhadores. Trab Educ Saúde. 2018;16(1):201–20. doi:10.1590/1981-7746-sol00097. [Portuguese]
73. Beltrão TA, Ramalho MNA, Barros MBSC, Silva FMC, Oliveira SHS. Acompanhamento de pessoas com câncer por enfermeiros da atenção primária. Rev Cubana Enferm. 2019;35(4):e3011. [Portuguese]
74. Castro RCL, Knauth DR. Papel dos atributos dos profissionais médicos na produção da abordagem centrada na pessoa em atenção primária à saúde. Cien Saude Colet. 2022;27(2):803–12. doi:10.1590/1413-81232022272.00392021. [Portuguese]
75. Dias EG. Adesão de idosos aos tratamentos da hipertensão arterial e as boas práticas de cuidado na perspectiva da integralidade [dissertação]. Ribeirão Preto (SP): Escola de Enfermagem de Ribeirão Preto, Universidade de São Paulo; 2018. [Portuguese]
76. Santos DVD, Freitas FO, Massuda A, Rodrigues CO, Stefanello S. Integração ensino-serviço na implantação de um curso de medicina no Paraná. Rev APS. 2022;25(Supl 1):90–108. [Portuguese]
77. Duarte ED, Silva KL, Tavares TS, Nishimot CLJ, Walty CMRF, Sena RR. Desafios do trabalho da enfermagem no cuidado às crianças com condições crônicas na atenção primária. Esc Anna Nery. 2015;19(4):648–55. doi:10.5935/1414-8145.20150087. [Portuguese]
78. Duayer MFF. Análise de implantação de um programa de gestão de casos no cuidado de idosos frágeis na Atenção Primária à Saúde [tese]. São Paulo (SP): Escola de Enfermagem, Universidade de São Paulo; 2018. [Portuguese]
79. Eslabão AD, Santos EO, Santos VCF, Rigatti R, Mello RM, Schneider JF. Saúde mental na estratégia saúde da família: caminhos para uma assistência integral em saúde. J Nurs Health. 2019;9(1):e199101. doi:10.15210/jonah.v9i1.11106. [Portuguese]
80. Facchini LA, Piccini RX, Tomasi E, Thumé E, Teixeira VA, Silveira DS, et al. Avaliação de efetividade da Atenção Básica à Saúde em municípios das regiões Sul e Nordeste do Brasil: contribuições metodológicas. Cad Saúde Pública. 2008;24(Suppl 1):S159–72. [Portuguese]
81. Fernandes L, Basílio N, Figueira S, Nunes JM. Saúde mental em Medicina Geral Familiar – obstáculos e expectativas percecionados pelos médicos de família. Cien Saude Colet. 2017;22(3):797–805. doi:10.1590/1413-81232017223.33212016. [Portuguese]
82. Fernández OM, Irarrázaval M, Ortega B, Carrasco P, Martínez P, Rojas G, et al. Salud mental primaria para niños, niñas y adolescentes institucionalizados: perspectiva de equipos de salud. Rev Latinoam Cienc Soc Niñez Juventud. 2021;19(2):1–19. doi:10.11600/rlcsnj.19.2.4175. [Spanish]
83. Ferreira ACS, Mattos M. Atenção multiprofissional ao idoso em condição crônica na Estratégia Saúde da Família. Rev Bras Promoç Saúde. 2018;31(3):1–10. doi:10.5020/18061230.2018.7576. [Portuguese]
84. Ferreira GS, Zanardo GLP, Moro LM, Damion M, Rocha KB. As práticas da psicologia no contexto interdisciplinar no NASF: uma revisão sistemática. Gerais: Rev Interinst Psicol. 2021;14(Suppl):e17386. doi:10.36298/gerais202114e17386. [Portuguese]
85. Ferreira RC, Chirelli MQ, Pereira AG. Abordagem psicológica na Atenção Básica em Saúde: da fragmentação à integralidade. Rev Bras Educ Med*.* 2011;35(2):177–85. doi:10.1590/S0100-55022011000200006. [Portuguese]
86. Figueiredo MD, Onocko Campos R. Saúde mental na atenção básica à saúde de Campinas, SP: uma rede ou um emaranhado? Cien Saude Colet. 2009;14(1):129–38. [Portuguese]
87. Foletto F, Aratani N. Análise da cobertura de saúde bucal e práticas das equipes de saúde bucal na atenção primária em municípios sul-mato-grossenses. Rev Odontol UNESP. 2022;51:e20220042. doi:10.1590/1807-2577.04222. [Portuguese]
88. Friestino JKO, Corrêa CRS, Moreira Filho DC. Percepções dos profissionais sobre o diagnóstico precoce do câncer infantojuvenil na atenção primária à saúde. Rev Bras Cancerol. 2017;63(4):265–72. [Portuguese]
89. Friestino JKO, Corrêa CRS, Souza AIJ, Fonsêca GS, Geremia AC, Moreira Filho DC. Qualificação profissional e o câncer infantojuvenil na atenção básica. Acta Paul Enferm. 2022;35:eAPE02771. doi:10.37689/acta-ape/2022AO02771. [Portuguese]
90. Galvão JR, Almeida PF, Santos AM, Bousquat A. Percursos e obstáculos na Rede de Atenção à Saúde: trajetórias assistenciais de mulheres em região de saúde do Nordeste brasileiro. Cad Saúde Pública. 2019;35(12):e00004119. doi:10.1590/0102-31100004119. [Portuguese]
91. Galvão JR, Almeida PF, Santos AM, Fernandes NFS. Trajetórias assistenciais de usuárias pela APS em uma região de saúde: trânsito livre, pontos de lentidão e parada. Physis Rev Saúde Coletiva. 2019;29(4):e290404. doi:10.1590/S0103-73312019290404. [Portuguese]
92. Gama CAP, Bicalho JMF, Dupin TO, Fonseca PC, Dias MEL, Moreira MFE. Estratégia de Saúde da Família e adesão ao tratamento do diabetes: fatores facilitadores. Rev Baiana Saúde Pública. 2021;45(1):11–35. doi:10.22278/2318-2660.2021.v45.n1.a3285. [Portuguese]
93. Gerlero S, Augsburger A, Duarte M, Gómez R, Yanco D. Salud mental y atención primaria: accesibilidad, integralidad y continuidad del cuidado en centros de salud, Argentina. Rev Argent Salud Pública. 2011;2(9):24–9. [Spanish]
94. Labegalini CMG, Aguirre HC, Peruzzo HE, Christinelli HCB, Souza RR, Marcon SS, et al. Atendimento de saúde à pessoas hipertensas e diabéticas: percepção de enfermeiros. Cienc Cuid Saude. 2022;21:e61580. doi:10.4025/ciencuidsaude.v21i0.61580. [Portuguese]
95. González CRA. A promoção da saúde como caminho para o envelhecimento ativo: o cuidado ao hipertenso em um centro de saúde escola [dissertação]. Rio de Janeiro (RJ): Escola Nacional de Saúde Pública Sergio Arouca, Fundação Oswaldo Cruz; 2008. [Portuguese]
96. Graever L. A assistência ao indivíduo com problemas relacionados ao uso de álcool e drogas ilícitas na Estratégia Saúde da Família [dissertação]. Rio de Janeiro (RJ): Escola Nacional de Saúde Pública Sergio Arouca, Fundação Oswaldo Cruz; 2013. [Portuguese]
97. Gurgel ALLG, Jorge MSB, Caminha ECCR, Maia Neto JP, Vasconcelos MGF. Cuidado em saúde mental na estratégia saúde da família: a experiência do apoio matricial. Rev Enferm UERJ. 2017;25:e7101. doi:10.12957/reuerj.2017.7101. [Portuguese]
98. Hirdes A. A perspectiva dos profissionais da Atenção Primária à Saúde sobre o apoio matricial em saúde mental. Cien Saude Colet. 2015;20(2):371–82. doi:10.1590/1413-81232015202.11122014. [Portuguese]
99. Hirdes A, Scarparo HBK. O labirinto e o minotauro: saúde mental na Atenção Primária à Saúde. Cien Saude Colet. 2015;20(2):383–93. doi:10.1590/1413-81232015202.12642013. [Portuguese]
100. Hirdes A, Silva MKR. Articulação entre apoio matricial em saúde mental e redes de atenção à saúde. Psicol Estud. 2017;22(3):383–94. doi:10.4025/psicolestud.v22i3.32579. [Portuguese]
101. Hoepfner C, Franco SC, Maciel RA, Hoepfner AMS. Programa de apoio matricial em cardiologia: qualificação e diálogo com profissionais da atenção primária. Saúde Soc. 2014;23(3):1091–101. doi:10.1590/S0104-12902014000300028. [Portuguese]
102. Hori AA, Nascimento AF. O Projeto Terapêutico Singular e as práticas de saúde mental nos Núcleos de Apoio à Saúde da Família (NASF) em Guarulhos (SP), Brasil. Cien Saude Colet. 2014;19(8):3561–71. doi:10.1590/1413-81232014198.11412013. [Portuguese]
103. Hsieh PL, Chen CM. Nurse-led care models in the context of community elders with chronic disease management: a systematic review. J Nurs (Taipei). 2016;63(4):35–49. doi:10.6224/JN.63.4.35. [Chinese (Traditional)]
104. Bolhari J, Ahmadkhaniha H, Hajebi A, Bagheri Yazdi SA, Naserbakht M, Karimi-Kisomi I, Tahmasebi S. Evaluation of mental health program integration into the primary health care system of Iran. Iran J Psychiatry Clin Psychol. 2012;17(4):271–8. [Persian]
105. Klein AP, d’Oliveira AFPL. O “cabo de força” da assistência: concepção e prática de psicólogos sobre o Apoio Matricial no Núcleo de Apoio à Saúde da Família. Cad Saúde Pública. 2017;33(1):e00158815. doi:10.1590/0102-311X00158815. [Portuguese]
106. Kolle AMM. Síntese de evidências para políticas de saúde: cuidado orientado para a família na Atenção Primária à Saúde [dissertação]. São Paulo (SP): Escola de Enfermagem, Universidade de São Paulo; 2019. [Portuguese]
107. Lage ÉG. Fatores associados a não efetivação da alta na atenção domiciliar para a Atenção Primária à Saúde [dissertação]. Belo Horizonte (MG): Universidade Federal de Minas Gerais, Escola de Enfermagem; 2018. [Portuguese]
108. Leal BMML. Movimentos do cuidado em saúde mental na Estratégia Saúde da Família [dissertação]. São Paulo (SP): Escola de Enfermagem, Universidade de São Paulo; 2010. [Portuguese]
109. Lemos SM. A inserção da saúde mental na atenção primária: um estudo qualitativo das práticas de profissionais na cidade de Manaus/AM [tese]. Rio de Janeiro (RJ): Universidade do Estado do Rio de Janeiro, Instituto de Medicina Social; 2019. [Portuguese]
110. Lieberenz LVA. Assistência à pessoa com condições crônicas na atenção primária à saúde [dissertação]. Belo Horizonte (MG): Universidade Federal de Minas Gerais, Escola de Enfermagem; 2020. [Portuguese]
111. Lima AIO, Severo AK, Andrade NL, Soares GP, Silva LM. O desafio da construção do cuidado integral em saúde mental no âmbito da atenção primária. Temas Psicol. 2013;21(1):71–82. doi:10.9788/TP2013.1-05. [Portuguese]
112. Lima AMJ. Contribuição da estruturação da Atenção Primária à Saúde segundo seus atributos essenciais para a qualidade da assistência em saúde mental: um estudo a partir do PMAQ-AB [tese]. Belo Horizonte (MG): Universidade Federal de Minas Gerais, Faculdade de Medicina; 2021. [Portuguese]
113. Liotti BCV. Atitudes dos profissionais da atenção primária frente à assistência aos usuários de álcool [dissertação]. Ribeirão Preto (SP): Escola de Enfermagem de Ribeirão Preto, Universidade de São Paulo; 2020. [Portuguese]
114. Santos JMM, Monteiro CN, Escrivão Junior Á. Rede de atenção à saúde no cuidado do paciente hipertenso, município de São Paulo, Brasil. Rev APS. 2021;24(2):321–37. doi:10.34019/1809-8363.2021.v24.16796. [Portuguese]
115. Medeiros CRG. Redes de atenção em saúde: o dilema dos pequenos municípios [tese]. Porto Alegre (RS): Universidade Federal do Rio Grande do Sul, Programa de Pós-Graduação em Enfermagem; 2013. [Portuguese]
116. Mendes SJ. Serviços farmacêuticos na Atenção Primária à Saúde: estudo etnográfico em serviços de saúde no município de São Paulo [tese]. São Paulo (SP): Faculdade de Ciências Farmacêuticas, Universidade de São Paulo; 2020. [Portuguese]
117. Minozzo F, Kammzetser CS, Debastiani C, Fait CS, Paulon SM. Grupos de saúde mental na atenção primária à saúde. Fractal Rev Psicol. 2012;24(2):323–40. doi:10.1590/S1984-02922012000200008. [Portuguese]
118. Morais APP. Saúde mental na atenção básica: o desafio da implementação do apoio matricial [tese]. São Paulo (SP): Faculdade de Saúde Pública, Universidade de São Paulo; 2010. [Portuguese]
119. Moretti-Pires RO, Campos DA. Equipe multiprofissional em Saúde da Família: do documental ao empírico no interior da Amazônia. Rev Bras Educ Med. 2010;34(3):379–89. [Portuguese]
120. Nascimento HG, Figueiredo AEB. O idoso com demência na atenção primária: revisão integrativa de literatura. Estud Interdiscipl Envelhec. 2018;23(2):51–71. [Portuguese]
121. Neves ACL. Estratégia Saúde da Família e pessoas com hipertensão e diabetes: redes sociais e longitudinalidade [dissertação]. Rio de Janeiro (RJ): Faculdade de Enfermagem, Universidade do Estado do Rio de Janeiro; 2019. [Portuguese]
122. Neves Filho JM. Saúde mental, atenção primária e Estratégia Saúde da Família: a implantação de unidades básicas de apoio à saúde mental na região sul do Município de São Paulo – um estudo de caso [dissertação]. São Paulo (SP): Faculdade de Saúde Pública, Universidade de São Paulo; 2009. [Portuguese]
123. Oliveira KS de, Baduy RS, Melchior R. O encontro entre o Núcleo de Apoio à Saúde da Família e as equipes de Saúde da Família: a produção de um coletivo cuidador. Physis. 2019;29(4):e290403. doi:10.1590/S0103-73312019290403. [Portuguese]
124. Oliveira PS, Santana FR, Gatto Júnior JR, Santos KS, Araujo PN, Fortuna CM. Matrix support in children’s mental health in Primary Health Care: institutional socio-clinical intervention research. Rev Esc Enferm USP. 2021;55:e03731. doi:10.1590/S1980-220X2020016803731. [Portuguese]
125. Oliveira TC de. Avaliação de desempenho do programa de controle do câncer de colo do útero: um modelo para aplicação local no município do Rio de Janeiro [dissertação]. Rio de Janeiro (RI): Programas de Pós-graduação da CAPES; 2010. [Portuguese]
126. Onocko-Campos RT, Campos GWS, Ferrer AL, Corrêa CRS, Madureira PR, Gama CAP, Dantas DV, Nascimento R. Avaliação de estratégias inovadoras na organização da Atenção Primária à Saúde. Rev Saúde Pública. 2012;46(1):43–50. doi:10.1590/S0034-89102012000100006. [Portuguese]
127. Padilha CS, Oliveira WF. Representação social do terapeuta comunitário na rede SUS. Ciênc Saúde Coletiva. 2013;18(8):2211–20. doi:10.1590/S1413-81232013000800014 . [Portuguese]
128. Paterra TSV. Avaliação do seguimento de mulheres com alterações no exame citopatológico do colo do útero [dissertação de mestrado]. Ribeirão Preto: Escola de Enfermagem de Ribeirão Preto, Universidade de São Paulo; 2018. 87 p. [Portuguese]
129. Pereira LF, Rech CR, Morini S. Autonomia e Práticas Integrativas e Complementares: significados e relações para usuários e profissionais da Atenção Primária à Saúde. Interface (Botucatu). 2021;25:e200079. doi:10.1590/Interface.200079. [Portuguese]
130. Pereira RMP, Amorim FF, Gondim MFN. A percepção e a prática dos profissionais da Atenção Primária à Saúde sobre a Saúde Mental. Interface (Botucatu). 2020;24(Supl 1):e190664. doi:10.1590/Interface.190664. [Portuguese]
131. Pérez-Cuevas R, Reyes Morales H, Doubova SV, Zepeda Arias M, Díaz Rodríguez G, Peña Valdovinos A, et al. Comprehensive diabetic and hypertensive patient care involving nurses working in family practice. Revista panamericana de salud pública. 2009;26(6):511–7. [Spanish]
132. Pinho LMG, Garcia VL, Nogueira-Martins MCF. Implantação da Residência Médica e Multiprofissional em Saúde da Família em um município paulista: percepção de residentes da primeira turma (2014-2016). Rev Bras Pesq Saúde. 2018;20(1):23–31. [Portuguese]
133. Pinto AGA, Jorge MSB, Vasconcelos MGF, Sampaio JJC, Lima GP, Bastos VC, Sampaio HAC. Apoio matricial como dispositivo do cuidado em saúde mental na atenção primária: olhares múltiplos e dispositivos para resolubilidade. Cien Saude Colet. 2012;17(3):653–60. [Portuguese]
134. Prado SRLA. Integralidade – um estudo a partir da atenção básica à saúde da criança em modelos assistenciais distintos [tese]. São Paulo (SP): Escola de Enfermagem da Universidade de São Paulo; 2005. [Portuguese]
135. Quinderé PHD, Jorge MSB, Franco TB. Rede de atenção psicossocial: qual o lugar da saúde mental? Physis. 2014;24(1):253–71. doi:10.1590/S0103-73312014000100014. [Portuguese]
136. Quinderé PHD, Jorge MSB, Nogueira MSL, Costa LFA, Vasconcelos MGF. Acessibilidade e resolubilidade da assistência em saúde mental: a experiência do apoio matricial. Cien Saude Colet. 2013;18(7):2157–66. [Portuguese]
137. Rampelotto GF, Schimith MD, Corcini LMCS, Garcia RP, Perlini NMOG. Ações educativas às pessoas com hipertensão e diabetes: trabalho do Agente Comunitário de Saúde rural. Rev Enferm UFSM. 2022;12:e43. doi:10.5902/2179769268715. [Portuguese]
138. Rezende CN, Abreu DMX, Lopes EAS, Santos AF, Machado ATGM. Coordenação do cuidado na Atenção Primária: gravidez, câncer de colo uterino e de mama como marcadores. Interface (Botucatu). 2022;26:e220060. doi:10.1590/interface.220060. [Portuguese]
139. Rigon E, Dalazen JVC, Busnello GF, Kolhs M, Olschowsky A, Kempfer SS. Experiências dos idosos e profissionais da saúde relacionadas ao cuidado pela estratégia saúde da família. Rev Enferm UERJ. 2016;24(5):e17030. doi:10.12957/reuerj.2016.17030. [Portuguese]
140. Rodrigues PM, Kostulski CA, Arpini DM. A construção de novas práticas na psicologia na atenção básica: a experiência de residentes psicólogos. Physis. 2021;31(2):e310215. doi:10.1590/S0103-73312021310215. [Portuguese]
141. Ruela LO, Moura CC, Gradim CVC, Stefanello J, Iunes DH, Prado RR. Implementação, acesso e uso das práticas integrativas e complementares no Sistema Único de Saúde: revisão da literatura. Cien Saude Colet. 2019;24(11):4239–50. doi:10.1590/1413-812320182411.06132018. [Portuguese]
142. Santos CM, Barbieri AR, Gonçalves CCM, Tsuha DH. Avaliação da rede de atenção ao portador de hipertensão arterial: estudo de uma região de saúde. Cad Saúde Pública. 2017;33(5):e00052816. doi:10.1590/0102-311X00052816. [Portuguese]
143. Santos ROM. O vínculo longitudinal como dispositivo do cuidado: Saúde da Família e doenças crônicas em uma comunidade do Rio de Janeiro [dissertação de mestrado]. Rio de Janeiro (RJ): Escola Nacional de Saúde Pública Sergio Arouca, Fundação Oswaldo Cruz; 2015. [Portuguese]
144. Santos ROM, Romano VF, Engstrom EM. Vínculo longitudinal na Saúde da Família: construção fundamentada no modelo de atenção, práticas interpessoais e organização dos serviços. Physis. 2018;28(2):e280206. doi:10.1590/S0103-73312018280206. [Portuguese]
145. Silva AP, Nascimento EGC, Pessoa Júnior JM, Melo JAL. “Por trás da máscara da loucura”: cenários e desafios da assistência à pessoa com esquizofrenia no âmbito da Atenção Básica. Fractal Rev Psicol. 2019;31(1):2–10. doi:10.22409/1984-0292/v31i1/5517. [Portuguese]
146. Silva ATC, Aguiar ME, Winck K, Rodrigues KGW, Sato ME, Grisi SJF, Brentani A, Rios IC. Núcleos de Apoio à Saúde da Família: desafios e potencialidades na visão dos profissionais da Atenção Primária do Município de São Paulo, Brasil. Cad Saúde Pública. 2012;28(11):2076–84. doi:10.1590/S0102-311X2012001100007. [Portuguese]
147. Silva G, Iglesias A, Dalbello-Araujo M, Badaró-Moreira MI. Práticas de cuidado integral às pessoas em sofrimento mental na Atenção Básica. Psicol Cienc Prof. 2017;37(2):404–17. doi:10.1590/1982-370301452015. [Portuguese]
148. Silva ROC. Apoio matricial em saúde mental: uma proposta de cuidado integral em saúde [dissertação de mestrado]. Niterói (RJ): Universidade Federal Fluminense, Escola de Enfermagem Aurora de Afonso Costa; 2018. [Portuguese]
149. Sousa SM. Contribuições do enfermeiro na integração dos cuidados no contexto das doenças crônicas não transmissíveis [tese]. Curitiba (PR): Universidade Federal do Paraná; 2019. [Portuguese]
150. Sousa SM, Bernardino E, Stegani MM, Utzumi FC, Aued GK, Maciel OB, et al. Configuração da rede de cuidados às doenças crônicas não transmissíveis na perspectiva da integração. Enferm Foco. 2022;13:e-202240. doi:10.21675/2357-707X.2022.v13.e-202240. [Portuguese]
151. Souza ECF, Vilar RLA, Rocha NSPD, Uchoa AC, Rocha PM. Acesso e acolhimento na atenção básica: uma análise da percepção dos usuários e profissionais de saúde. Cad Saúde Pública. 2008;24(Suppl 1):S100–10. doi:10.1590/S0102-311X2008001300017. [Portuguese]
152. Souza GRM, Cazola LHO, Pícoli RP. Atuação do enfermeiro da Atenção Primária à Saúde na assistência oncológica: revisão integrativa. Cogitare Enferm. 2018;23(4):e58152. doi:10.5380/ce.v23i4.58152. [Portuguese]
153. Souza JB, Manorov M, Martins EL, Reis L, Buss ITS, Heidemann RTS. Itinerários terapêuticos das mulheres com câncer de mama: percepções dos enfermeiros da atenção primária em saúde. Rev Fun Care Online. 2021;13:1186–92. doi:10.9789/2175-5361.rpcfo.v13.9239. [Portuguese]
154. Souza TT, Calvo MCM. Avaliabilidade dos Núcleos de Apoio à Saúde da Família com foco na integração às equipes apoiadas. Rev Saude Publica. 2018;52:41. doi:10.11606/S1518-8787.2018052000122. [Portuguese]
155. Sow A, Criel B, Branger B, Roland M, De Spiegelaere M. Expérience d´intégration de la santé mentale en première ligne de soins en Guinée. Pan Afr Med J. 2020;37:107. doi:10.11604/pamj.2020.37.107.20351. [French]
156. Taniguchi TG. (Des)enCAPSulando: os agentes comunitários de saúde e o cuidado da pessoa com transtorno mental [dissertação de mestrado]. São Paulo (SP): Universidade de São Paulo, Faculdade de Saúde Pública; 2018. [Portuguese]
157. Tatmatsu DB, Araújo ACC. Atenção primária e saúde mental: contribuições e potencialidades do apoio matricial. Mudanças Psicol Saúde. 2016;24(2):71–9. [Portuguese]
158. Tavares MFL, Mendonça MHM, Rocha RM. Práticas em saúde no contexto de reorientação da atenção primária no Estado do Rio de Janeiro, Brasil, na visão das usuárias e dos profissionais de saúde. Cad Saúde Pública. 2009;25(5):1054–62. doi:10.1590/S0102-311X2009000500015. [Portuguese]
159. Tesser CD, Sousa IMC, Nascimento MC. Práticas Integrativas e Complementares na Atenção Primária à Saúde brasileira. Saude Debate. 2018;42(Spe 1):174–88. doi:10.1590/0103-11042018S112. [Portuguese]
160. van Stralen CJ, Belisário SA, van Stralen TBS, Lima AMD, Massote AW, Oliveira CL. Percepção dos usuários e profissionais de saúde sobre atenção básica: comparação entre unidades com e sem saúde da família na Região Centro-Oeste do Brasil. Cad Saúde Pública. 2008;24(Suppl 1):S148–58. doi:10.1590/S0102-311X2008001300017. [Portuguese]
161. Vello LS. Saúde do Trabalhador na Atenção Primária à Saúde: estudo de caso em um município na região metropolitana de São Paulo [dissertação de mestrado]. São Paulo (SP): Universidade de São Paulo, Faculdade de Saúde Pública; 2015. [Portuguese]
162. Venancio SI, Rosa TEC, Bersusa AAS. Atenção integral à hipertensão arterial e diabetes mellitus: implementação da Linha de Cuidado em uma Região de Saúde do estado de São Paulo, Brasil. Physis. 2016;26(1):113–35. doi:10.1590/S0103-73312016000100008. [Portuguese]
163. Viana DM, Lima AF. Saúde mental e atenção primária: compreendendo articulações e práticas na saúde da família no Ceará. Rev Psicol (Fortaleza). 2016;7(2):118–30. [Portuguese]
164. Vieira ADFP, Gomes LO, Moraes CF, Nóbrega OT. Capacitação, conhecimentos e crenças de médicos da Atenção Primária à Saúde relacionados ao envelhecimento. Rev Kairós Gerontol. 2019;22(1):329–52. doi:10.23925/2176-901X.2019v22i1p329-352. [Portuguese]
165. Vieira RR. Reflexões multidisciplinares em serviço e sua influência na prática do médico da atenção básica: contribuições da educação permanente em saúde [dissertação de mestrado]. Niterói (RJ): Universidade Federal Fluminense, Escola de Enfermagem Aurora de Afonso Costa; 2018. [Portuguese]
166. Wiik MCG. Atenção básica no cuidado à Hipertensão Arterial Sistêmica: a voz dos usuários do SUS [dissertação de mestrado]. São Paulo (SP): Universidade de São Paulo, Faculdade de Saúde Pública; 2022. [Portuguese]
