# Supplementary material for: Understanding the role and organization of health workers delivering non-communicable disease management in primary care in low- and middle-income countries: a scoping review
Source: BMC Prim Care. 2025 Nov 17;26:365. doi: 10.1186/s12875-025-03033-3 (PMC12625573; doi:10.1186/s12875-025-03033-3)
Supplement: Supplementary file 5 — Additional file 5. [file 12875_2025_3033_MOESM5_ESM.docx]

**ADDITIONAL FILE 4**

Appendix S4: Publications by country, categorized by WHO region and income classification

| Income classification | Sum of # of citations | % of Total citations |
| --- | --- | --- |
| lower-middle-income | 66 | 38% |
| low-income | 6 | 3% |
| upper-middle-income | 103 | 59% |
| Grand Total | 175 | 100% |

**American Samoa transitioned from a middle-income country to a high-income country in 2022 but was included in the review, given it met the criteria at the time of study publication*

***Multi-country study*

Appendix S5: Publications by country, categorized by WHO region and income classification

| WHO Region | Sum of # of citations | % of Total citations |
| --- | --- | --- |
| African Region | 47 | 27% |
| Eastern Mediterranean Region | 13 | 7% |
| European Region | 4 | 2% |
| Region of the Americas | 43 | 25% |
| South-East Asian Region | 34 | 19% |
| Western Pacific Region | 34 | 19% |
| Grand Total | 175 | 100% |

**American Samoa transitioned from a middle-income country to a high-income country in 2022 but was included in the review, given it met the criteria at the time of study publication;*

***Includes multi-country study, American Samoa and Tajikistan*

Appendix S6: Distribution of Citations by Health Conditions by Income Classification and MDT Presence

| Health condition/  Income classification | # of citations  with MDT | % of citations  with MDT |
| --- | --- | --- |
| **NCD** | 84 | 67% |
| Lower Income | 1 | 1% |
| Lower Middle Income | 25 | 30% |
| Upper Middle Income | 58 | 69% |
| **MH and NCD-MH** | 41 | 33% |
| Lower Income | 3 | 2% |
| Lower Middle Income | 21 | 17% |
| Upper Middle Income | 17 | 14% |
| Total | 125 | 100% |
