# Supplementary material for: Understanding the role and organization of health workers delivering non-communicable disease management in primary care in low- and middle-income countries: a scoping review
Source: BMC Prim Care. 2025 Nov 17;26:365. doi: 10.1186/s12875-025-03033-3 (PMC12625573; doi:10.1186/s12875-025-03033-3)
Supplement: Supplementary file 6 — Additional file 6. [file 12875_2025_3033_MOESM6_ESM.pdf]

## Appendix S7: Characteristics of included studies: health worker cadres and roles in NCD management

| Title | Lead author | Year of Publ. | Country | Conditions | Generalist MD | Generalist Services | Specialist MD | Specialist Services | Nurse | Nurse Services | CHW | CHW Services | Lay HW [volunteers] | Lay HW Services | Other | Do MDTs support NCD manage ment? |
|---|---|---|---|---|---|---|---|---|---|---|---|---|---|---|---|---|
| Study on the determinants of health professionals' performance on diabetes management care in China | Shanshan Jing | 2023 | China | Type 2 diabetes | Yes | Screening, Education and Counselling, Diagnosis, Linkage to Care, Treatment and Management, Referral | N/A | N/A | No | N/A | No | N/A | No | N/A | No | No |
| The Vietnam Multicomponent Collaborative Care for Depression Program: Development of Depression Care for Low- and Middle-Income Nations | Victoria K. Ngo, PhD | 2014 | Vietnam | Depression | Yes | Diagnosis, Treatment and Management | No | N/A | Yes | Screening, Treatment and Management, Education and Counselling | Yes | Education and Counselling, Follow-Up | No | N/A | No | Yes |
| Determinants of an integrated cervical cancer screening services in primary healthcare: sharing lessons from Kisumu, Kenya | Eyo Akpan | 2023 | Kenya | Cervical Cancer | No | N/A | No | N/A | Yes | Screening, Treatment and Management | No | N/A | Yes | Linkage to Care | No | Yes |
| Ability and Preparedness of Family Physicians to Recognise and Treat Adolescent Mental Health Disorders in Nigeria and Ghana | Tijani Idris Ahmad Oseni | 2023 | Nigeria and Ghana | Adolescent mental health | Yes | Diagnosis, Treatment and Management | N/A | N/A | N/A | N/A | N/A | N/A | N/A | N/A | No | No |
| Analysis of patient medication compliance and quality of life of physician-pharmacist collaborative clinics for T2DM management in primary healthcare in China: A mixed-methods study | Jie Xiao | 2023 | China | Type 2 diabetes | Yes | Diagnosis, Treatment and Management | No | N/A | No | N/A | No | N/A | No | N/A | Pharmacist | Yes |
| Integrating Mental Health Services into the Primary Health Care System: The Need for Reform in Iran | Hajebi | 2021 | Iran | Mental health disorders (depression or anxiety disorder, victims of domestic violence, patients at risk for suicide, psychosis, bipolar disorder) | Yes | Screening, Diagnosis, Treatment and Management, Referral | No | N/A | No | N/A | Yes | Screening, Follow-Up, Linkage to Care | No | N/A | Clinical psychologist, social workers | Yes |
| Specialized Outpatient Services: Community Mental Health Centers (CMHCs) | Vandad Sharifi | 2021 | Iran | Common mental disorders (depressive and anxiety disorders) and severe mental disorders (schizophrenia, schizoaffecti | Yes | Screening, Diagnosis, Treatment and Management, Home Visits, Appointment Reminders, Referral | Yes | Screening, Education and Counselling, Treatment and Management, Home Visits | Yes | Education and Counselling, Telephone Follow-Up, Treatment Adherence, Home Visits | No | N/A | No | N/A | Clinical psychologist, social worker | Yes |

| | | | | | | | | | | | | | | | | |
|---|---|---|---|---|---|---|---|---|---|---|---|---|---|---|---|---|
| | | | | ve, and bipolar disorders) | | | | | | | | | | | | |
| Implementation process and outcomes of a mental health programme integrated in primary care clinics in rural Mexico: a mixed-methods study | Georgina Miguel-Esponda | 2020 | Mexico | Mood disorders - depression, anxiety, bipolar disorder | Yes | Screening, Diagnosis, Treatment and Management, Follow-Up, Referral | No | N/A | No | N/A | Yes | Screening, Education and Counselling, Treatment Adherence, Appointment Reminders | No | N/A | No | Yes |
| Mental health services provided by medical officers in primary health centres in Kolar district in Karnataka, India: A situational assessment | Gautham Melur Sukumar | 2019 | India | mental health disorders | Yes | Diagnosis, Treatment and Management, Referral | N/A | N/A | N/A | N/A | N/A | N/A | N/A | N/A | No | No |
| "We just have to help": Community health workers' informal task-shifting and task-sharing practices for hypertension and diabetes care in Nigeria | Whenayon Simeon Ajisegi | 2023 | Nigeria | Hypertension and diabetes | No | N/A | N/A | N/A | No | N/A | Yes | Patient Registration, Screening, Treatment and Management, Education and Counselling, Home Visits, Medication Adherence, Linkage to Care (community resources), Referral, Follow-Up | No | N/A | N/A | No |
| Transforming care for patients living with diabetes in rural Mexico: a qualitative study of patient and provider experiences and perceptions of shared medical appointments | Martha de Lourdes Arrieta-Canales | 2023 | Mexico | Diabetes | Yes | Screening, Diagnosis, Treatment and Management, Follow-Up | No | N/A | Yes | Education and Counselling | Yes | Education and Counselling | No | N/A | No | Yes |
| Dementia detection practice among primary care practitioners: A cross-sectional study in Hulu Langat District, Selangor | Norhayati Aziz | 2023 | Malaysia | Dementia | Yes | Diagnosis, Treatment and Management, Referral | No | N/A | Yes | Diagnosis, Treatment and Management | No | N/A | No | N/A | "other allied professionals" | Yes |
| Impact of the family doctor system on the continuity of care for diabetics in urban China: a difference-in-difference analysis | Xinyi Liu | 2023 | China | Diabetes | Yes | Education and Counselling, Screening, Diagnosis, Treatment and Management, Referral | No | N/A | Yes | N/A | No | N/A | No | N/A | No | Yes |
| Exploring barriers to dementia screening and management services by general practitioners in China: a qualitative study using the COM-B model | Ni Gong, Dan Yang | 2023 | China | Dementia | Yes | Screening, Diagnosis, Treatment and Management | N/A | N/A | No | N/A | No | N/A | No | N/A | No | No |
| Effectiveness of a provider and patient-focused intervention to improve hypertension management and control in the primary health care setting in Cuba: a controlled before-after study | Esteban LondoÃ±o Agudelo | 2023 | Cuba | Hypertension | Yes | Screening, Diagnosis, Education and Counselling, Treatment and Management, Appointment Reminders | No | N/A | Yes | Education and Counselling, Screening, Diagnosis, Treatment and Management, Appointment Reminders | No | N/A | No | N/A | Psychologists, nutritionists, physiotherapists | Yes |
| Physicians' and nurses' perspective on chronic disease care practices in Primary Health Care in Brazil: a qualitative study | Carolinny Nunes Oliveira | 2022 | Brazil | Hypertension, Diabetes mellitus | Yes | Screening, Diagnosis, Treatment and Management, | No | N/A | Yes | Screening, Education and Counselling, Proactive Case Detection, | Yes | Proactive Case Detection | No | N/A | dentists | Yes |

| | | | | | | | | | | | | | | | | |
|---|---|---|---|---|---|---|---|---|---|---|---|---|---|---|---|---|
| | | | | | | Follow-Up, Referral | | | | Linkage to Care, Referral | | | | | | |
| Collaborative care compared to enhanced standard treatment of depression with co-morbid medical conditions among patients from rural South India: a cluster randomized controlled trial (HOPE Study) | Krishnamachari Srinivasan | 2022 | India | Major depressive disorder, dysthymia, generalized anxiety disorder, and/or panic disorder on the MINI-International Neuropsychiatric Interview, co-diagnosed with hypertension, diabetes, and/or ischemic heart disease | Yes | Screening, Diagnosis, Treatment and Management, Referral | Yes | Not specified | Yes | Screening, Diagnosis, Treatment and Management, Referral, Education and Counselling | Yes | Screening, Education and Counselling, Appointment Reminders | No | N/A | Pharmacist, counsellor | Yes |
| Lessons Learnt From Pilot Cervical Cancer Screening and Treatment Programmes Integrated to Routine Primary Health Care Services in Benin, Cote d'Ivoire, and Senegal | Farida Selmouni | 2022 | Benin, Cote d'Ivoire, and Senegal | Cervical Cancer | Yes | Screening, Diagnosis, Treatment and Management, Referral | Yes (gynecologist) | Screening, Treatment and Management | Yes | Screening, Diagnosis, Treatment and Management | Yes | Education and Counselling | No | N/A | Midwives | Yes |
| Effectiveness of integrated care for older adults with depression and hypertension in rural China: A cluster randomized controlled trial | Shulin Chen | 2022 | China | Hypertension and depression | Yes | Screening, Education and Counselling, Management and Treatment, Referral, Follow-Up | Yes | Diagnosis, Treatment and Management | No | N/A | Yes | Not specified | Yes (Aging Workers) | Home Visits, Education and Counselling, Treatment Adherence | No | Yes |
| Measuring the impact of community-based interventions on type 2 diabetes control during the COVID-19 pandemic in Cape Town - A mixed methods study | Neal J. David | 2022 | South Africa | Type 2 diabetes | No | N/A | No | N/A | No | N/A | Yes | Screening, Home Visits, Linkage to Care, Medication Delivery | No | N/A | No | No |
| Trained health extension workers correctly identify high blood pressure in rural districts of Northwest Ethiopia: a diagnostic accuracy study | Destaw Fetene Teshome | 2022 | Ethiopia | Hypertension | No | N/A | No | N/A | Yes | Home Visits, Screening, Education and Counselling, Linkage to Care | Yes (Health Extension Worker) | Home Visits, Screening, Education and Counselling, Linkage to Care | No | N/A | N/A | No |
| Psychiatric patients' experience in receiving services provided by Non-specialist community nurses, southern Thailand | Saifon Aekwarangkoon | 2022 | Thailand | major depressive disorder and schizophrenia | N/A | N/A | N/A | N/A | Yes | Education and Counselling, Treatment and Management, Linkage to Care (community resources) | N/A | N/A | N/A | N/A | No | No |
| Acceptability and fidelity of a psychosocial intervention (PROACTIVE) for older adults with depression in a basic health unit in Sao Paulo, Brazil: a qualitative study | Maiara Garcia Henrique | 2021 | Brazil | Depression | "Other team member" | N/A | No | N/A | Yes | N/A | Yes | N/A | No | N/A | "Other team member" | Yes |
| Strengthening management of Non-communicable diseases in primary care, Malawi: A short report | Amos Mailosi | 2021 | Malawi | Hypertension, Diabetes, | Yes | Diagnosis, Treatment and Management, Education and Counselling, Referral | No | N/A | No | N/A | No | N/A | No | N/A | Pharmacy Technician, Medical Assistant | No |

| | | | | | | | | | | | | | | | | |
|---|---|---|---|---|---|---|---|---|---|---|---|---|---|---|---|---|
| Perceptions of ASHA workers in the HOPE collaborative care mental health intervention in rural South India: a qualitative analysis | Stuti Bansal | 2021 | India | depression, anxiety, comorbid with diabetes or cardiovascular disease | No | N/A | No | N/A | No | N/A | Yes | Education and Counselling, Screening, Linkage to Care, Appointment Reminders | No | N/A | No | Yes |
| Mental health capacity building in Mali by training rural general practitioners and raising community awareness | Oumar Poudiougou | 2021 | Mali | Mental disorders (pychosis, mood disorders, anxiety disorders, post-traumatic stress disorder (PTSD), addictions, autism and dementia.) | Yes | Education and Counselling, Diagnosis, Treatment and Management, Referral | No | N/A | No | N/A | No | N/A | No | N/A | No | Yes |
| Evaluation and pilot implementation of essential interventions for the management of hypertension and prevention of cardiovascular diseases in primary health care in the Republic of Tajikistan | Dylan Collins | 2021 | Republic of Tajikistan | hypertension and cardiovascular disease | Yes | Screening, Education and Counselling, Treatment and Management | No | N/A | Yes | Education and Counselling, Screening, Treatment and Management | No | N/A | No | N/A | No | Yes |
| Management of chronic kidney disease: perspectives of Brazilian primary care physicians | Thatiane Delatorre | 2021 | Brazil | Chronic Kidney Disease (CKD) | Yes | Screening, Diagnosis, Education and Counselling, Linkage to Care, Treatment and Management, Referral, Follow-Up | No | N/A | Yes | Home Visits | Yes | Home Visits | No | N/A | No | Yes |
| Early Experiences in the Integration of Non-communicable Diseases into Emergency Primary Health Care, Beni Region, Democratic Republic of the Congo | RUWAN RATNAYAKE | 2021 | Democratic Republic of the Congo | hypertension and diabetes (type 1 and 2) | No | N/A | No | N/A | No | N/A | Yes | Education and Counselling, Follow-Up, Treatment Adherence, Referral | No | N/A | No | Yes |
| Effectiveness of a task-sharing collaborative care model for identification and management of depressive symptoms in patients with hypertension attending public sector primary care clinics in South Africa: pragmatic parallel cluster randomised controlled | Inge Petersen | 2021 | South Africa | Depression, Hypertension | Yes | Treatment and Management | No | N/A | Yes | Screening, Referral | No | N/A | Yes (lay mental health counsellors) | Education and Counselling | No | Yes |
| Improving primary care for diabetes and hypertension: findings from implementation research in rural South India | Dorothy Lall | 2020 | India | diabetes and hypertension | Yes | Screening, Treatment and Management | No | N/A | Yes | Screening, Education and Counselling | Yes - ASHAs | Home Visits, Appointment Reminders, Follow-Up | No | N/A | Pharmacist, Lab Tech | Yes |
| Community health workers for Non-communicable disease prevention and control in Nepal: a qualitative study | Lal B Rawal | 2020 | Nepal | Hypertension, diabetes | No | N/A | No | N/A | No | N/A | Yes | Screening, Diagnosis, Education and Counselling, Treatment and Management, Referral | Yes | Education and Counselling, Linkage to Care | No | No |
| Team-based primary health care for Non-communicable diseases: complexities in South India | Dorothy Lall | 2020 | India | Diabetes and cardiovascular disease | Yes | Screening, Treatment and Management | No | N/A | Yes | Screening, Education and Counselling | No | N/A | No | N/A | Pharmacist, Lab Tech | Yes |

| | | | | | | | | | | | | | | | | |
|---|---|---|---|---|---|---|---|---|---|---|---|---|---|---|---|---|
| Hypertension Care Coordination and Feasibility of Involving Female Community Health Volunteers in Hypertension Management in Kavre District, Nepal: A Qualitative Study | Jingru Tan | 2020 | Nepal | Hypertension | No | N/A | No | N/A | No | N/A | No | N/A | Yes | Screening, Education and Counselling, Referral | Pharmacist | Yes |
| Process, quality and challenges of diabetes care in primary care: a study of district health network in Thailand | Jumnean Somanawat | 2020 | Thailand | Diabetes | No | N/A | No | N/A | Yes | Treatment and Management, Education and Counselling | No | N/A | Yes | Screening, Education and Counselling | Public Health officer, Dental Nurse | Yes |
| Bridging the mental health treatment gap: effects of a collaborative care intervention (matrix support) in the detection and treatment of mental disorders in a Brazilian city | Sonia Saraiva | 2020 | Brazil | Mental disorders including anxiety and depressive disorders. | Yes | Screening, Diagnosis, Treatment and Management | No | N/A | Yes | N/A | No | N/A | No | N/A | No | Yes |
| Effect of collaborative care between traditional and faith healers and primary health-care workers on psychosis outcomes in Nigeria and Ghana (COSIMPO): a cluster randomised controlled trial | Oye Gureje | 2020 | Nigeria and Ghana | Psychosis | Yes | Education and Counselling, Treatment and Management, Referral | N/A | N/A | N/A | N/A | N/A | N/A | N/A | N/A | Traditional Faith Healers | Yes |
| Evaluating the integration of chronic care elements in primary health care for people with mental illness: a longitudinal study in Nepal conducted among primary health care workers | Nawaraj Upadhaya | 2020 | Nepal | Mental illness: depression, epilepsy, psychosis, and alcohol use disorder | Yes | Screening, Diagnosis, Education and Counselling, Treatment and Management | No | N/A | Yes | Education and Counselling | Yes | N/A | Yes | Proactive Case Detection, Referral | No | Yes |
| Strengthening human and physical infrastructure of primary healthcare settings to deliver hypertension care in Vietnam: a mixed-methods comparison of two provinces | Lana Meiqari | 2020 | Vietnam | Hypertension | Yes | Diagnosis, Linkage to Care, Treatment and Management, Referral | No | N/A | Yes | role Not specified | No | N/A | No | N/A | Pharmacist and Midwife (roles Not defined) | Yes |
| Diabetes care kNowledge and practice among primary care physicians in Southeast Nigeria: a cross-sectional study | Ejiofor Ugwu | 2020 | Nigeria | Diabetes | Yes | Screening, Diagnosis, Treatment and Management, Education and Counselling, Treatment Adherence, Referral | N/A | N/A | No | N/A | No | N/A | No | N/A | No | No |
| Incorporating the patient-centered approach into clinical practice helps improve quality of care in cases of hypertension: a retrospective cohort study | Nida Buawangpong | 2020 | Thailand | hypertension | Yes | Screening, Diagnosis, Education and Counselling, Treatment and Management, Referral | No | N/A | Yes | Screening | No | N/A | No | N/A | No | Yes |
| Adapting a nurse-led primary care initiative to cardiovascular disease control in Ghana: a qualitative study | Leah A. Haykin | 2020 | Ghana | Cardiovascular disease | Yes | N/A | No | N/A | Yes | Screening, Diagnosis, Education and Counselling, Treatment and management, Referral, Home Visits | No | N/A | No | N/A | No | No |
| Outcomes of a primary care mental health implementation program in rural Rwanda: A quasi-experimental implementation-effectiveness study | Stephanie L. Smith | 2020 | Rwanda | mental disorders (bipolar, epilepsy, psychosis, | No | N/A | No | N/A | Yes | Screening, Diagnosis, Treatment and Management, Education and Counselling, | Yes | Proactive Case Detection, Treatment Adherence, Education and Counselling | No | N/A | No | Yes |

| | | | | | | | | | | | | | | | | |
|---|---|---|---|---|---|---|---|---|---|---|---|---|---|---|---|---|
| | | | | schizophrenia, others) | | | | | | Linkage to Care (community resources), Referral | | | | | | |
| Observational stepped-wedge analysis of a community health worker-led intervention for diabetes and hypertension in rural Mexico | Devin T Worster | 2020 | Mexico | Diabetes and hypertension | Yes | Treatment and Management | No | N/A | No | N/A | Yes | Home Visits, Treatment Adherence, Education and Counselling, Other: Supporting active case retention | No | N/A | No | Yes |
| Community-, facility-, and individual-level outcomes of a district mental healthcare plan in a low-resource setting in Nepal: A population-based evaluation | Mark J. D. Jordans | 2019 | Nepal | depression, psychosis, alcohol use disorder (AUD), and epilepsy | Yes | Screening, Diagnosis, Treatment and Management, Education and Counselling | No | N/A | Yes | Education and Counselling | No | N/A | Yes | Proactive Case Detection, Home Visits | Auxiliary Health Workers and Health Assistants | Yes |
| Can General Practitioners manage mental disorders in primary care? A partially randomised, pragmatic, cluster trial | Sabrina Gabrielle Anjara | 2019 | Indonesia | Mental health (Depressive episode, Panic disorder, OCD, Social Phobia, Agora Phobia, Specific (Isolated) Phobia, Generalized Anxiety Disorder, Mixed Anxiety Depression) | Yes | Screening, Diagnosis, Treatment and Management, Education and Counselling, Referral | Yes | Treatment and Management | Yes | Screening, Diagnosis, Treatment and Management, Education and Counselling, Referral | No | N/A | No | N/A | Clinical psychologist | Yes |
| What do Accredited Social Health Activists need to provide comprehensive care that incorporates Non-communicable diseases? Findings from a qualitative study in Andhra Pradesh, India | Marwa Abdel-All | 2019 | India | Cardiovascular Disease, Diabetes, Cancer and Stroke | No | N/A | No | N/A | No | N/A | Yes (ASHAs) | Education and Counselling, Screening, Diagnosis, Linkage to Care, Treatment and Management, Referral | No | N/A | No | Yes |
| Evaluation of a collaborative care model for integrated primary care of common mental disorders comorbid with chronic conditions in South Africa | Inge Petersen | 2019 | South Africa | Mental disorders comorbid with chronic conditions (depressive and alcohol use disorder (AUD) symptoms) | Yes | Treatment and Management | No | N/A | Yes | Screening, Diagnosis, Referral | No | N/A | Yes (lay mental health counsellors) | Education and Counselling | No | Yes |
| Challenges in primary care for diabetes and hypertension: an observational study of the Kolar district in rural India | Dorothy Lall | 2019 | India | diabetes and hypertension | Yes | Screening, Diagnosis, Education and Counselling, Treatment and | No | N/A | No | N/A | No | N/A | No | N/A | No | Yes |

| | | | | | | | | | | | | | | | | |
|---|---|---|---|---|---|---|---|---|---|---|---|---|---|---|---|---|
| | | | | | | Management, Referral | | | | | | | | | | |
| Development and feasibility assessment of a collaborative stepped care intervention for management of depression in the mental health in primary care (MeHPriC) project, Lagos, Nigeria | Abiodun O. Adewuya1 | 2019 | Nigeria | Depression | Yes | Screening, Diagnosis, Treatment and Management, Education and Counselling, Referral | N/A | N/A | Yes | Screening, Diagnosis, Treatment and Management, Education and Counselling, Referral | Yes | Screening, Education and Counselling | N/A | N/A | N/A | Yes |
| Integrating a diabetes and hypertension case management package within primary health care: a mixed methods feasibility study in Bangladesh | R Huque | 2018 | Banglades h | Diabetes and hypertension | Yes | Diagnosis | No | N/A | Yes | Education and Counselling | No | N/A | No | N/A | No | Yes |
| The impact of an intervention to improve diabetes management in primary healthcare professionals' practices in Brazil | Michelly Georgia da Silva Marinho | 2017 | Brazil | type 2 diabetes | Yes | Education and Counselling, Screening, Diagnosis, Linkage to Care (active patient search in case of missed appt), Treatment and Management, Treatment adherence, Referral | No | N/A | Yes | Education and Counselling, Screening, Diagnosis, Linkage to Care (active patient search in case of missed appt), Treatment and Management, Referral | Yes | N/A | No | N/A | N/A | Yes |
| Collaborative Care model in mental health. Scope and experiences after three years of activity in Mexico City | Valerio Villamil-Salcedo | 2017 | Mexico | diabetes, hypertension , obesity, depressive and anxiety disorders | Yes | Screening, Treatment and Management, Follow-Up | Yes - psychiatris t | Screening, Diagnosis, Treatment and Management | Yes | Patient Registration | No | N/A | No | N/A | Social Worker, Psychologist, Dentist | No |
| Task shifting to improve the provision of integrated chronic care: realist evaluation of a lay health worker intervention in rural South Africa | Felix Limbani | 2019 | South Africa | Hypertension | No | N/A | No | N/A | Yes | role Not specified | No | N/A | Yes | Screening, Patient Registration, Education and Counselling, Appointment Reminders | No | Yes |
| Mental health kNowledge, attitudes, and self-efficacy among primary care physicians working in the Greater Tunis area of Tunisia | Jessica Spagnolo | 2018 | Tunisia | Mental health including anxiety, depression, alcohol use disorders, drug use disorders, psychosis (including schizophreni a), suicide/self-harm | Yes | Diagnosis, Education and Counselling, Treatment and Management, Referral | N/A | N/A | N/A | N/A | N/A | N/A | N/A | N/A | No | No |
| Physician-community pharmacist collaborative care in diabetes management: a pilot study | Bouchra Bakr Mouhtadi | 2018 | Lebanon | Type 2 Diabetes | Yes | Diagnosis, Referral | No | N/A | No | N/A | No | N/A | No | N/A | pharmacist | Yes |
| Can lay health workers support the management of hypertension? Findings of a cluster randomised trial in South Africa | Jane Goudge | 2018 | South Africa | Hypertension | No | N/A | No | N/A | Yes | Junior nurse - Screening. Professional nurse - Diagnosis, | No | N/A | Yes | Patient Registration, Education and Counselling, Screening, | No | Yes |

| | | | | | | | | | | | | | | | | |
|---|---|---|---|---|---|---|---|---|---|---|---|---|---|---|---|---|
| | | | | | | | | | | Treatment and Management | | | | Appointment Reminders | | |
| New model for diabetes primary health care based on patient empowerment and the right to preventive health: the MIDE program | Margarita Blanco-Cornejo | 2017 | Mexico | Diabetes | Yes | Diagnosis, Treatment and Management, Referral | No | N/A | Yes | Education and Counselling | No | N/A | No | N/A | Dietician, Social Worker, Dentist, Psychologist, Physical Trainer | Yes |
| Perceptions of health managers and professionals about mental health and primary care integration in Rio de Janeiro: a mixed methods study | Karen AthiÃ© | 2016 | Brazil | Mental health | Yes | Education and Counselling, Screening, Diagnosis, Linkage to Care, Treatment and Management, Referral | Yes | Not specified | Yes | Not specified | Yes | Not specified | No | N/A | Psychologist (role not specified) | Yes |
| Effect of a Primary Care-Based Psychological Intervention on Symptoms of Common Mental Disorders in Zimbabwe: A Randomized Clinical Trial | Dixon Chibanda | 2016 | Zimbabwe | Common mental disorders (depression and anxiety) | No | N/A | Yes | Education and Counselling, Treatment and Management, Referral, Appointment Reminder | No | N/A | No | N/A | Yes | Education and Counselling, Linkage to Care, Treatment and Management, Referral, Appointment Reminders, Telephone Follow-Up | Health Promotion Officer (supervisor) | Yes |
| Can integrated health services delivery have an impact on hypertension management? A cross-sectional study in two cities of China | Haitao Li | 2016 | China | Hypertension | Yes | Screening, Diagnosis, Education and Counselling, Linkage to Care, Treatment and Management, Referral | No | N/A | Yes | role Not specified | No | N/A | No | N/A | No | No |
| Recommendations from primary care providers for integrating mental health in a primary care system in rural Nepal | Bibhav Acharya | 2016 | Nepal | Mental Health Conditions | Yes | Screening, Diagnosis, Treatment and Management, Referral | No | N/A | No | N/A | Yes | N/A | No | N/A | Auxiliary Health Workers and Health Assistants | Yes |
| Effectiveness of multidisciplinary intervention on blood pressure control in primary health care: a randomized clinical trial | Regina Kuhmmer | 2016 | Brazil | Hypertension | No | Education and Counselling | No | N/A | No | N/A | No | N/A | No | N/A | physical educators, pharmacists, dieticians | Yes |
| Implementation of 'matrix support' (collaborative care) to reduce asthma and COPD Referall and improve primary care management in Brazil: a pilot observational study | Sonia Maria Martins | 2016 | Brazil | Asthma and chronic obstructive pulmonary disease (COPD) | Yes | Screening, Diagnosis, Education and Counselling, Treatment and Management, Referral | Yes | Not specified | Yes | Screening, Diagnosis, Education and Counselling, Treatment and Management, Referral | No | N/A | No | N/A | Pharmacist and chest therapist (role not specified) | Yes |
| Assessing the impact of general practitioner team service on perceived quality of care among patients with Non-communicable diseases in China: a natural experimental study | JIA YIN | 2016 | China | Hypertension, diabetes, and hyperlipidemia | Yes | Education and Counselling, Screening, Diagnosis, Treatment and Management, Referral | No | N/A | Yes | Patient Registration, Treatment and Management, Education and Counselling | No | N/A | No | N/A | No | Yes |
| Providers' kNowledge, attitudes, and practices related to colorectal cancer control in Brazil | Douglas M. Puricelli Perin | 2015 | Brazil | Colorectal cancer (CRC) | Yes | Screening | Maybe? | N/A | Yes | Screening | No | N/A | No | N/A | No | Yes |

| | | | | | | | | | | | | | | | | |
|---|---|---|---|---|---|---|---|---|---|---|---|---|---|---|---|---|
| Primary health care nurses' management practices of common mental health conditions in KwaZulu-Natal, South Africa | Faith N. Dube | 2015 | South Africa | common mental health conditions | N/A | N/A | N/A | N/A | Yes | Diagnosis, Treatment and Management | N/A | N/A | N/A | N/A | No | No |
| Care for post-stroke patients at Malaysian public health centres: self-reported practices of family medicine specialists | Aznida F Abdul Aziz | 2014 | Malaysia | stroke | Yes | Education and Counselling, Treatment and Management, Referral | N/A | N/A | No | N/A | No | N/A | No | N/A | No | No |
| The impact of a lay counselor led collaborative care intervention for common mental disorders in public and private primary care: a qualitative evaluation nested in the MANAS trial in Goa, India | Sachin Shinde | 2013 | India | Depressive or anxiety disorders (common mental disorders or CMD) | Yes | Screening, Education and Counselling, Treatment and Management, Referral | Yes | Treatment and Management, Referral | No | N/A | No | N/A | Yes | Treatment and Management, Education and Counselling, Treatment Adherence | No | Yes |
| 'Opening up the mind': problem-solving therapy delivered by female lay health workers to improve access to evidence-based care for depression and other common mental disorders through the Friendship Bench Project in Zimbabwe | Melanie Abas | 2016 | Zimbabwe | Depression and other common mental disorders (CMDs) | No | N/A | Yes | Treatment and Management | No | N/A | No | N/A | Yes | Screening, Education and Counselling, Linkage to Care, Treatment and Management, Referral | Health Promotion Officer (supervisor) | Yes |
| Pharmaceutical care issues identified by pharmacists in patients with diabetes, hypertension or hyperlipidaemia in primary care settings | Siew Siang Chua | 2012 | Malaysia | Diabetes, Hypertension, Hyperlipidemia | Yes | Treatment and Management | No | N/A | Yes | Education and Counselling | No | N/A | No | N/A | Pharmacist and Dietician | Yes |
| The Efficiency of Mental Health Integration in Primary Health Care: a Ten-year Study | Reza Khadivi | 2012 | Iran | Mental disorders: 1- Severe mental disorders, such as major depression, schizophrenia, bipolar disorders, etc., 2 - Mild mental disorders, such as neurosis, anxiety, etc., 3 - Convulsive disorders and 4 - Behavioral disorders | Yes | Screening, Diagnosis, Treatment and Management | No | N/A | No | N/A | No | N/A | No | N/A | No | No |
| Lay health worker led intervention for depressive and anxiety disorders in India: impact on clinical and disability outcomes over 12 months | Vikram Patel | 2011 | India | Depressive and anxiety disorders (common mental disorders) | Yes | Screening, Diagnosis, Education and counselling, Treatment and Management, Referral | Yes (psychiatrist) | Treatment and Management | No | N/A | No | N/A | Yes (lay health counsellor) | Treatment and Management | No | Yes |
| Problem-solving therapy for depression and common mental disorders in Zimbabwe: piloting a task-shifting primary mental health | Dixon Chibanda | 2011 | Zimbabwe | depression and common mental disorders in a | No | N/A | Yes | Treatment and Management | No | N/A | No | N/A | Yes | Education and Counselling, Home Visits | Clinical psychologist | Yes |

| | | | | | | | | | | | | | | | | |
|---|---|---|---|---|---|---|---|---|---|---|---|---|---|---|---|---|
| care intervention in a population with a high prevalence of people living with HIV | | | | population with a high prevalence of people living with HIV | | | | | | | | | | | | |
| Effectiveness of an intervention led by lay health counsellors for depressive and anxiety disorders in primary care in Goa, India (MANAS): a cluster randomised controlled trial | Vikram Patel | 2010 | India | Depressive and anxiety disorders (the so-called common mental disorders). | Yes | Screening, Diagnosis, Education and counselling, Treatment and Management, Referral | Yes (psychiatrist) | Treatment and Management | No | N/A | No | N/A | Yes (lay health counsellor) | Treatment and Management | No | Yes |
| Management of chronic kidney disease: primary health-care setting, self-care and multidisciplinary approach | A.M. Cueto-ManzaNo | 2010 | Mexico | CKD - At High Risk<br>For the present analysis, 51 (28%) patients with overweight/obesity (Non-diabetics, Non-hypertensives), 45 (25%) with hypertension (Non-diabetics) and 88 (47%) with Type 2 diabetes mellitus (DM2) (with or without hypertension) were studied prospectively. | Yes | Education and Counselling, Treatment and Management | No | N/A | No | N/A | No | N/A | No | N/A | Social Worker, Dietician, Physical Trainer | Yes |
| Audit on cardiovascular disease preventive care in general practice | Chan S C | 2008 | Malaysia | Cardiovascular disease | Yes | Screening | No | N/A | No | N/A | No | N/A | No | N/A | No | No |
| Noncommunicable disease management in resource-poor settings: a primary care model from rural South Africa | R. Coleman | 1998 | South Africa | Hypertension, Diabetes, Asthma, and Epilepsy | Yes | Screening, Diagnosis, Treatment and Management | No | N/A | Yes | Screening, Diagnosis, Treatment and Management, Education and Counselling | No | N/A | No | N/A | No | Yes |
| Integrating psychiatry into primary care: an experimental model | J.D. Adeyemi | 1994 | Nigeria | Psychiatric disease | Yes | Diagnosis | N/A | N/A | N/A | N/A | N/A | N/A | N/A | N/A | No | No |
| Effectiveness of a Non-physician community health-care provider-led intensive blood pressure intervention versus usual care on cardiovascular disease (CRHCP): an open-label, blinded-endpoint, cluster-randomised trial | Jiang He | 2023 | China | Hypertension | Yes | N/A | No | N/A | No | N/A | No | N/A | No | N/A | No | No |
| InNovative mobile-health led participatory approach to comprehensive screening and treatment of diabetes (IMPACT diabetes): rationale, design, and baseline characteristics | Abhinav Bass | 2023 | India | Diabetes | Yes | Diagnosis, Treatment and Management, | No | N/A | No | N/A | Yes (ASHAs) | Screening, Referral, Home Visits, Follow-Up, | No | N/A | No | Yes |

| | | | | | | | | | | | | | | | | |
|---|---|---|---|---|---|---|---|---|---|---|---|---|---|---|---|---|
| | | | | | | Education and Counselling | | | | | | Appointment Reminders, Education and Counselling | | | | |
| Community-based rehabilitation intervention for people with schizophrenia in Ethiopia (RISE): results of a 12-month cluster-randomised controlled trial | Laura Asher | 2022 | Ethiopia | Schizophrenia | Yes (Health Officer) | Treatment and Management, Education and Counselling, Referral | No | N/A | Yes | Treatment and Management, Education and Counselling | Yes | Education and Counselling, Treatment and Management, Home Visits, Treatment Adherence | No | N/A | Yes - Community-Based Rehab | Yes |
| The capacity of primary health care centers in Jordan to manage hypertension: areas for improvement | Omar Al-Hadeethi | 2022 | Jordan | Hypertension | Yes | Screening, Diagnosis, Education and Counselling, Treatment and Management, Referral | No | N/A | Yes | role Not specified | No | N/A | No | N/A | Pharmacist, Lab Tech | Yes |
| Quality of Actions to Control Cervical Cancer in Bahia, Brazil | Eduarda Ferreira dos Anjos | 2021 | Brazil | Cervical cancer | Yes | Treatment and Management, Education and Counselling | No | N/A | Yes | Treatment and Management, Education and Counselling | Yes | Patient Registration | No | N/A | students | Yes |
| SMART Mental Health Project: process evaluation to understand the barriers and facilitators for implementation of multifaceted intervention in rural India | Abha Tewari | 2021 | India | common mental disorders (CMDs) (which included depression, increased suicide risk and emotional stress) | Yes | Diagnosis, Treatment and Management, Education and Counselling, Follow-Up | No | N/A | No | N/A | Yes | Screening, Proactive Case Detection, Follow-Up, Treatment adherence, Education and Counselling | No | N/A | No | Yes |
| Contribution of community health workers to primary health care performance in Brazil | Alaneir de Fatima dos Santos | 2020 | Brazil | Women's and children's health, diabetes, and hypertension | Yes | N/A | No | N/A | Yes | Not specified | Yes | Home Visits, Screening, Proactive Case Detection, Linkage to Care | No | N/A | other professionals | Yes |
| Decentralising NCD management in rural southern Africa: evaluation of a pilot implementation study | Ashley Sharp | 2020 | Eswatini | Type II diabetes and hypertension | No | N/A | No | N/A | Yes | Screening, Diagnosis, Education and Counselling, Treatment and Management, Referral | No | N/A | No | N/A | No | No |
| Challenges and lessons from a primary care intervention in a Brazilian municipality | Monica V Andrade | 2019 | Brazil | hypertension and diabetes | Yes | Screening, Diagnosis, Treatment and Management, Education and Counselling | No | N/A | Yes | role Not specified | Yes | Patient Registration, Home Visits | No | N/A | No | Yes |
| Challenges in delivery and performance of a cervical cancer prevention program in the Kurdistan Region of the Iraq health system | Vian H. Rasul | 2018 | Iraq | cervical cancer | Yes | Screening | Yes | Screening | Yes | Screening | No | N/A | No | N/A | No | Yes |
| Quality of care for Non-communicable diseases in the Republic of Moldova: a survey across primary health care facilities and pharmacies | Carolyn Blake | 2019 | Republic of Moldova | Hypertension, ischemic heart disease, and diabetes | Yes | Screening, Diagnosis, Treatment and Management, Education and | No | N/A | Yes | role Not specified | No | N/A | No | N/A | Medical Assistants | No |

| | | | | | | | | | | | | | | | | |
|---|---|---|---|---|---|---|---|---|---|---|---|---|---|---|---|---|
| | | | | | | Counselling, Referral | | | | | | | | | | |
| Impact of training primary care physicians in behavioral counseling to reduce cardiovascular disease risk factors in Ecuador | Manuel E. BaldeÃ³n | 2018 | Ecuador | Cardiovascular disease (CVD) | Yes | Education and Counselling, Treatment and Management, Referral | No | N/A | No | N/A | No | N/A | No | N/A | No | No |
| Impact of different approaches of primary care mental health on the prevalence of mental disorders | Leonardo Moscovici | 2018 | Brazil | mental or emotional disorders (MED) | Yes | Screening, Education and Counselling, Treatment and Management | Yes | Screening, Education and Counselling, Treatment and Management | Yes | Screening, Education and Counselling, Treatment and Management | Yes | Home Visits | No | N/A | Mental Health Specialists | Yes |
| Assessment of hypertension chronic care model: Pacic application in Bosnia and Herzegovina | Natasa Pilipovic-Broceta | 2018 | Bosnia and Herzegovina | Hypertension | Yes | Screening, Diagnosis, Education and Counselling, Treatment and Management | N/A | N/A | No | N/A | No | N/A | No | N/A | No | No |
| The adoption of roles by primary care providers during implementation of the new chronic disease guidelines in urban Mongolia: A qualitative study | Oyun Chimeddamba | 2016 | Mongolia | Hypertension and Diabetes | Yes | Screening, Diagnosis, Education and Counselling, Treatment and Management, Referral | No | N/A | Yes | Screening, Education and Counselling | No | N/A | No | N/A | No | Yes |
| Barefoot therapists: Barriers and facilitators to delivering maternal mental health care through peer volunteers in Pakistan: A qualitative study | Najia Atif | 2016 | Pakistan | Perinatal depression | No | N/A | No | N/A | No | N/A | No | N/A | Yes | Home Visits, Education and Counselling | No | Yes |
| KNowledge and practice of PHC physicians toward the detection and management of hypertension and other CVD risk factors in Egypt | Mostafa A. Abolfotouh | 2011 | Egypt | Hypertension and other CVD risk factors | Yes | Screening, Treatment and Management | N/A | N/A | Yes | Counselling | No | N/A | No | N/A | N/A | No |
| Type 2 diabetes management in nurse-led primary healthcare settings in urban and rural Cameroon | Andre Pascal Kengne | 2009 | Cameroon | Type 2 diabetes | Yes | Study focused on nurses' role | No | N/A | Yes | Education and Counselling, Treatment and Management, Follow-Up | No | N/A | No | N/A | No | Yes |
| Primary health care for hypertension by nurses in rural and urban sub-Saharan Africa | Andre P. Kengne | 2009 | Cameroon | Hypertension | Yes | Study focused on nurses' role | No | N/A | Yes | Diagnosis, Education and Counselling, Treatment and Management, Follow-Up | No | N/A | No | N/A | No | No |
| Implementation of Chronic Care Model for Diabetes Self-Management: A Quantitative Analysis | Rashid M. Ansari | 2022 | Pakistan | Type 2 diabetes | Yes | Treatment and Management | No | N/A | Yes | Education and Counselling | No | N/A | No | N/A | No | Yes |
| "We find what we look for, and we look for what we kNow": factors interacting with a mental health training program to influence its expected outcomes in Tunisia | Jessica SpagNolo | 2018 | Tunisia | Mental health including depression, psychosis, self-harm/suicide, and alcohol/drug use disorders | Yes | Screening, Diagnosis, Treatment and Management, Education and Counselling, Referral, Telephone Follow-Up | Yes | Not specified | Yes | Not specified | No | N/A | No | N/A | Psychologist, Social Worker | Yes |
| Building primary care practitioners' attitudes and confidence in mental health skills in a post-conflict society: a Cambodian example | David C. Henderson | 2005 | Cambodia | Mental health | Yes | Screening, Diagnosis, Education and Counselling, Treatment and | Yes | Not specified | N/A | N/A | N/A | N/A | N/A | N/A | No | No |

| | | | | | | | | | | | | | | | | |
|---|---|---|---|---|---|---|---|---|---|---|---|---|---|---|---|---|
| | | | | | | Management (Traditional Medicine) | | | | | | | | | | |
| Work process and mental health care flow in primary health care | Luana Cristina Bellini Cardoso | 2020 | Brazil | mental disorders | Yes | Education and Counselling, Screening, Diagnosis, Treatment and Management, Referral, Follow-Up | No | N/A | Yes | Referral | Yes | Proactive Case Detection, Home Visits | No | N/A | psychologist | Yes |
| A integralidade das praticas dos medicos cubanos No Programa Mais Medicos na cidade do Rio de Janeiro, Brasil | Cassiano Mendes Franco | 2018 | Brazil | mental disorders, diabetes, hypertension | Yes | Education and Counselling, Screening, Diagnosis, Treatment and Management, Referral, Linkage to Care | No | N/A | Yes | Not specified | Yes | Not specified | No | N/A | No | Yes |
| Mental health care technologies: Primary Care practices and processes | Daniella Barbosa Campos | 2018 | Brazil | mental disorders | Yes | Treatment and Management | No | N/A | Yes | Patient Registration, Screening, Referral, Appointment Reminders, Treatment and Management | No | N/A | No | N/A | No | Yes |
| Work in primary health care: a comprehensive mental health | Juliana Reale Cacapava | 2009 | Brazil | Mental health | Yes | Education and Counselling, Referral | No | N/A | Yes | role Not specified | No | N/A | No | N/A | Social Worker, Psychologist | Yes |
| Monitoring of cervical cancer control actions and associated factors | Eduarda Ferreira dos Anjos | 2021 | Brazil | Cervical cancer | Yes | Screening, Treatment and Management | No | N/A | Yes | Education, Screening, Treatment and Management, Referral | Yes | Not specified | No | N/A | No | Yes |
| Early detection of chronic renal disease: coordinated work between primary and specialized care in an ambulatory renal network of Peru | Jessica Bravo-Zuniga | 2018 | Peru | Chronic renal disease | Yes | Screening, Diagnosis, Treatment and Management | No | N/A | Yes | role Not specified | No | N/A | No | N/A | Nutritionists and psychologists (roles not defined) | Yes |
| Pharmaceutical Care in Primary Care: an Experience with Hypertensive Patients in the North of Brazil | Ilvia Silva Gomes | 2022 | Brazil | Hypertension | No | N/A | No | N/A | No | N/A | No | N/A | No | N/A | pharmacist | Yes |
| The effectiveness and acceptability of mobile telephone adherence support for management of depression in the Mental Health in Primary Care (MeHPriC) project, Lagos, Nigeria: A pilot cluster randomised controlled trial | Adewuya | 2019 | Nigeria | Mental health (depression) | Yes | Treatment and Management, Referral | No | N/A | No | N/A | Yes | Education and Counselling, Follow-up | No | N/A | No | Yes |
| The effect of the BATHE interview technique on the empowerment of diabetic patients in primary care: A cluster randomised controlled study | Akturan | 2017 | Turkey | Type 2 diabetes mellitus | Yes | Education and counselling; Treatment and management | No | N/A | No | N/A | No | N/A | No | N/A | No | No |
| Effectiveness of a targeted and tailored pharmacist-led intervention to improve adherence to antihypertensive drugs among patients with type 2 diabetes in Indonesia: A cluster randomised controlled trial | Alfian | 2021 | Indonesia | Type 2 diabetes | No | N/A | No | N/A | No | N/A | No | N/A | No | N/A | Pharmacist | No |
| Process Evaluation of Implementing a Pharmacist-Led Intervention to Improve Adherence to Antihypertensive Drugs Among Patients with Type 2 Diabetes in Indonesian Community Health Centers | Alfian | 2021 | Indonesia | Type 2 Diabetes and Hypertension | No | N/A | No | N/A | No | N/A | No | N/A | No | N/A | Pharmacists | No |

| | | | | | | | | | | | | | | | | |
|---|---|---|---|---|---|---|---|---|---|---|---|---|---|---|---|---|
| The impact of intensified clinical care on glycaemic control in patients with type 2 diabetes at Khayelitsha Community Health Centre, South Africa: Quasi-experimental study | Allerton | 2020 | South Africa | Type 2 diabetes | Yes | Treatment and Management, Diagnosis, Referral | No | N/A | Yes | Screening, Treatment and Management | No | N/A | No | N/A | Health Promoter | Yes |
| mHealth to Train Community Health Nurses in Visual Inspection With Acetic Acid for Cervical Cancer Screening in Ghana | Asgary | 2016 | Ghana | Cervical cancer | No | N/A | Yes | Screening, Diagnosis, Treatment and Management | Yes | Screening, Diagnosis, Referral, Education and Counselling | No | N/A | No | N/A | No | Yes |
| Screen and Triage by Community Extension Workers to Facilitate Screen and Treat: Task-Sharing Strategy to Achieve Universal Coverage for Cervical Cancer Screening in Nigeria | Awolude | 2018 | Nigeria | cervical cancer | Yes | Screening | No | N/A | Yes | Screening | Yes | Screening | No | N/A | Nurse midwives | Yes |
| The effectiveness of diabetes medication therapy adherence clinic to improve glycaemic control among patients with type 2 diabetes mellitus: a randomised controlled trial | Chai | 2020 | Malaysia | Type 2 diabetes mellitus | Yes | Screening, Diagnosis, Treatment and Management | No | N/A | Yes | Screening | No | N/A | No | N/A | pharmacist | Yes |
| Impact of an educational intervention in primary care on fasting blood glucose levels and diabetes kNowledge among patients with type 2 diabetes mellitus in rural China | Chen | 2020 | China | Type 2 diabetes mellitus | Yes | Diagnosis, Education and Counselling, Treatment and Management, Referrals | Yes | Diagnosis, Counselling | Yes | Not specified | No | N/A | No | N/A | No | Yes |
| The impact of community health educators on uptake of cervical and breast cancer prevention services in Nigeria | Chigbu | 2017 | Nigeria | Cervical and breast cancer | No | N/A | No | N/A | Yes | Screening | No | Education and Counselling | No | N/A | No | No |
| Effects of pharmacist-led patient education on diabetes-related kNowledge and medication adherence: A home-based study | Chow | 2015 | Malaysia | Diabetes | No | N/A | No | N/A | No | N/A | No | N/A | No | N/A | Pharmacist | No |
| A partnership-oriented and culturally-sensitive communication style of doctors can impact the health outcomes of patients with chronic illnesses in Indonesia | Claramita | 2020 | Indonesia | Hypertension , Type-2 Diabetes Mellitus (T2DM) | Yes | Education and counselling, Treatment and management | No | N/A | No | N/A | No | N/A | No | N/A | No | No |
| Nurse-community health worker team improves diabetes care in American Samoa: results of a randomized controlled trial | DePue | 2013 | American Samoa | Diabetes | No | N/A | No | N/A | Yes | Education and Counselling | No | N/A | No | N/A | No | No |
| Impact Assessment of Pharmaceutical Care in the Management of Hypertension and Coronary Risk Factors after Discharge | Cazarim | 2016 | Brazil | Hypertension | No | N/A | No | N/A | No | N/A | No | N/A | No | N/A | Pharmacist | Yes |
| Efficacy of an education course delivered to community health workers in diabetes control: A randomized clinical trial | deSouza | 2017 | Brazil | Type 2 diabetes mellitus | No | N/A | No | N/A | No | N/A | Yes | Education and Counselling, Other - home visits | No | N/A | No | Yes |
| Using Community Health Workers and a Smartphone Application to Improve Diabetes Control in Rural Guatemala | Duffy | 2020 | Guatemala | Type 2 diabetes | No | N/A | No | N/A | No | N/A | Yes | Screening, Diagnosis, Treatment and Management, Education and Counselling, Referral | No | N/A | No | No |
| Educational Outreach with an Integrated Clinical Tool for Nurse-Led Non-communicable Chronic Disease Management in Primary Care in South Africa: A Pragmatic Cluster Randomised Controlled Trial | Fairall | 2016 | South Africa | Hypertension , diabetes, chronic respiratory disease or those who screened positive for depression. | No | N/A | No | N/A | Yes | Education and counselling; Screening; Diagnosis; Treatment and management | No | N/A | No | N/A | No | No |
| Impact of pharmacist counseling on health-related quality of life of patients with type 2 | Fajriansyah | 2020 | Indonesia | Type 2 Diabetes | No | N/A | No | N/A | No | N/A | No | N/A | No | N/A | pharmacist | No |

| | | | | | | | | | | | | | | | | |
|---|---|---|---|---|---|---|---|---|---|---|---|---|---|---|---|---|
| diabetes mellitus: a cluster randomized controlled study | | | | Mellitus (T2DM) | | | | | | | | | | | | |
| Feasibility of a community-based cervical cancer screening with "test and treat" strategy using self-sample for an HPV test: Experience from rural Cameroon, Africa | Domgue | 2019 | Cameroon | cervical cancer, HPV | No | N/A | No | N/A | Yes | Education and Counselling, Diagnosis, Referral | Yes | Education and Counselling | No | N/A | Yes | Yes |
| Impact evaluation of a healthy lifestyle intervention to reduce cardiovascular disease risk in health centers in San Jose, Costa Rica and Chiapas, Mexico | Fort | 2015 | Costa Rica and Mexico | CVD, hypertension, diabetes, hyperlipidemia | Yes | Not specified | No | N/A | Yes | N/A | No | N/A | No | N/A | Health Promoters, Nutritionist | Yes |
| An Educational Intervention to Improve Statin Use: Cluster RCT at the Primary Care Level in Argentina | Gulayin | 2019 | Argentina | hypercholesterolemia among patients with moderate to high CVD risk. | Yes | Education and Counselling, Treatment and Management, Other - Diagnosis and monitoring | No | N/A | No | N/A | No | N/A | No | N/A | Pharmacist assistants | Yes |
| Feasibility and efficacy of nurse-led team management intervention for improving the self-management of type 2 diabetes patients in a Chinese community: a randomized controlled trial | Guo | 2019 | China | Type 2 Diabetes | Yes | Not specified | Yes | Not specified | Yes | Education and Counselling, Referral, Follow-Up, Other - home visit, telephone consultation | No | N/A | No | N/A | No | Yes |
| Training nurses in task-shifting strategies for the management and control of hypertension in Ghana: a mixed-methods study | Gyamfi | 2017 | Ghana | hypertension | No | N/A | No | N/A | Yes | Screening, Diagnosis, Referral, Treatment and Management | No | N/A | No | N/A | No | No |
| Lifestyle interventions for hypertension treatment among Iranian women in primary health-care settings: Results of a randomized controlled trial | N/A | 2015 | Iran | Hypertension | Yes | Treatment and Management | No | N/A | Yes | Education and Counselling | No | N/A | No | N/A | No | Yes |
| A Community-Based Intervention for Managing Hypertension in Rural South Asia | Jafar | 2020 | Bangladesh, Pakistan, and Sri Lanka | hypertension | Yes | Treatment and Management | Yes | Other – health status monitoring | No | N/A | Yes | Education and Counselling, Appointment reminders, Screening, Referral | No | N/A | No | Yes |
| Effectiveness of a nurse-led multidisciplinary self-management program for patients with coronary heart disease in communities: A randomized controlled trial | Jiang | 2020 | China | Coronary heart disease (CHD) | Yes | Screening, Diagnosis, Treatment and Management, Education and Counselling, Referral | Yes | Treatment and Management, Education and Counselling | Yes | Education and Counselling, Diagnosis, Treatment and Management, Telephone Follow-up | No | N/A | No | N/A | No | Yes |
| Setting-up nurse-led pilot clinics for the management of Non-communicable diseases at primary health care level in resource-limited settings of Africa | Kengne | 2010 | Cameroon | Asthma, type 2 diabetes mellitus, epilepsy and hypertension | Yes | Not specified | No | N/A | Yes | Not specified | No | N/A | No | N/A | No | No |
| Effectiveness of the Chronic Care Model in Type 2 Diabetes Management in a Community Health Service Center in China: A Group Randomized Experimental Study | Kong | 2019 | China | Type 2 Diabetes | Yes | Education and Counselling | No | N/A | No | N/A | No | N/A | No | N/A | Public Health Assistants | Yes |
| A COPD health management program in a community-based primary care setting: a randomized controlled trial | Lou | 2015 | China | COPD | Yes | Treatment and Management, Diagnosis, Follow-Up, | Yes | Treatment and Management, Diagnosis, Follow-Up | No | N/A | No | N/A | No | N/A | No | Yes |

| | | | | | | | | | | | | | | | | |
|---|---|---|---|---|---|---|---|---|---|---|---|---|---|---|---|---|
| | | | | | | Other - Home visits | | | | | | | | | | |
| Effectiveness of a group diabetes education programme in under-served communities in South Africa: a pragmatic cluster randomized controlled trial | Mash | 2014 | South Africa | Type 2 diabetes | No | N/A | No | N/A | No | N/A | No | N/A | Yes | Education and Counselling | No | No |
| Nursing case management for people with hypertension in primary health care: A randomized controlled trial | Da Silva | 2020 | Brazil | Hypertension | Yes | Not specified | Yes | Not specified | No | N/A | No | N/A | No | N/A | N/A | Yes |
| Increasing use of mental health services in remote areas using mobile technology: a pre-post evaluation of the SMART Mental Health project in rural India | Maulik | 2017 | India | Depression and anxiety | Yes | Diagnosis, Treatment and Management, Referral | No | N/A | No | N/A | Yes | Screening, treatment, management, Referral, follow-up, Other – home visits, treatment adherence | No | N/A | No | Yes |
| Evaluation of a pilot hypertension management programme for Guatemalan adults | Montano | 2015 | Guatemala | Hypertension | Yes | Treatment and Management | No | N/A | Yes | Screening, Diagnosis, Education and Counselling, referral, Nurses follow-up | Yes | Education and Counselling | No | N/A | No | Yes |
| Physicians and pharmacists: collaboration to improve the quality of prescriptions in primary care in Mexico | MiNo-Leon | 2012 | Mexico | Diabetes, Hypertension | No | N/A | No | N/A | No | N/A | No | N/A | No | N/A | Pharmacists | Yes |
| Prevention of diabetes in rural India with a telemedicine intervention | Mohan | 2012 | India | Diabetes | No | Not specified | No | N/A | No | N/A | No | N/A | Yes | Education and Counselling, Screening, | No | No |
| Point-of-care testing improves diabetes management in a primary care clinic in South Africa | Motta | 2017 | South Africa | diabetes | No | N/A | No | N/A | Yes | Screening, Other – Follow-up | No | N/A | No | N/A | No | Yes |
| Development of a community participation program for diabetes mellitus prevention in a primary care unit, Thailand | Oba | 2011 | Thailand | PRE-diabetes mellitus | Yes | N/A | No | N/A | Yes | Treatment and management | No | N/A | No | N/A | No | Yes |
| Economic evaluation of a pharmaceutical care program for elderly diabetic and hypertensive patients in primary health care: a 36-month randomized controlled clinical trial | Obreli-Neto | 2015 | Brazil | Diabetes, Hypertension | Yes | N/A | No | N/A | Yes | Education and Counselling, Diagnosis | No | N/A | No | N/A | No | Yes |
| Health insurance coverage with or without a nurse-led task shifting strategy for hypertension control: A pragmatic cluster randomized trial in Ghana | Ogedegbe | 2018 | Ghana | hypertension | Yes | N/A | No | N/A | Yes | Diagnosis, Referral, Education and Counselling, Follow Up | No | N/A | No | N/A | No | No |
| Outcomes and costs of implementing a community-based intervention for hypertension in an urban slum in Kenya | Oti | 2016 | Kenya | hypertension | No | N/A | No | N/A | No | N/A | No | N/A | No | N/A | No | Yes |
| Population-level scale-up of cervical cancer prevention services in a low-resource setting: development, implementation, and evaluation of the cervical cancer prevention program in Zambia | Parham | 2015 | Zambia | cervical cancer | Yes | N/A | No | N/A | Yes | Treatment and Management, Referral | Yes | Education and Screening, Other - community mobilization, health promotion and adherence efforts | Yes | Education and Counselling, Referral, Other - health promotion | No | No |
| Association of Multifaceted Mobile Technology-Enabled Primary Care Intervention With Cardiovascular Disease Risk Management in Rural Indonesia | Patel | 2019 | Indonesia | Cardiovascular disease | Yes | N/A | No | N/A | Yes | Treatment and Management | Yes | Screening, Counselling and Education, Referral, Other - Data collection | No | N/A | No | Yes |

| | | | | | | | | | | | | | | | | |
|---|---|---|---|---|---|---|---|---|---|---|---|---|---|---|---|---|
| The Healthy Activity Program (HAP), a lay counsellor-delivered brief psychological treatment for severe depression, in primary care in India: a randomised controlled trial | Patel | 2017 | India | Severe depression | Yes | Treatment and management | Yes | Treatment and management | No | N/A | No | N/A | No | N/A | No | Yes |
| SMARThealth India: A stepped-wedge, cluster randomised controlled trial of a community health worker managed mobile health intervention for people assessed at high cardiovascular disease risk in rural India | Peiris | 2019 | India | CVDs | Yes | Treatment and management, Diagnosis | Yes | Treatment and management, Diagnosis | No | N/A | Yes | Screening, Education and Counselling, Referral, follow-up visits, | No | N/A | No | Yes |
| The impact of a face-to-face peer-support intervention on adults with type 2 diabetes: a cluster-randomised trial | Pienaar | 2021 | South Africa | Type 2 diabetes | Yes | N/A | No | N/A | No | N/A | Yes | Education and Counselling, Follow up | No | N/A | No | Yes |
| Effects of 2 educational interventions on the management of hypertensive patients in primary health care | Pimenta | 2014 | Brazil | Hypertension | Yes | N/A | No | N/A | No | N/A | No | N/A | No | N/A | No | No |
| Reduction of cardiovascular risk in patients with metabolic syndrome in a community health center after a pharmaceutical care program of pharmacotherapy follow-up | Plaster | 2012 | Brazil | Diabetes, Hypertension, high cardiovascular risk/metabolic syndrome | No | N/A | No | N/A | No | N/A | No | N/A | No | N/A | No | Yes |
| Enhanced care by community health workers in improving treatment adherence to antidepressant medication in rural women with major depression | Pradeep | 2014 | India | Depression | Yes | Diagnosis, Treatment and Management | No | N/A | No | N/A | Yes | Education and Counselling, Follow-up | No | N/A | No | Yes |
| Long-term glycaemic outcome of structured nurse-led diabetes care in rural Africa | Price | 2011 | South Africa | Diabetes mellitus, type 2 | No | N/A | Yes | Not specified | No | N/A | No | N/A | Yes | Education and Counselling | No | No |
| Cognitive behaviour therapy-based intervention by community health workers for mothers with depression and their infants in rural Pakistan: a cluster-randomised controlled trial | Rahman | 2008 | Pakistan | Maternal depression | No | N/A | No | N/A | No | N/A | No | N/A | Yes | Education and Counselling | No | No |
| An innovative approach to improve the detection and treatment of risk factors in poor urban settings: a feasibility study in Argentina | Rosana | 2021 | Argentina | CVDs | Yes | Treatment and Management, Other- Educational outreach visits | No | N/A | Yes | Screening | Yes | Education and Counselling, Follow-up, Other - schedule a medical appointment | No | N/A | No | Yes |
| An NGO-Implemented Community-Clinic Health Worker Approach to Providing Long-Term Care for Hypertension in a Remote Region of Southern India | Sankaran | 2017 | India | hypertension | Yes | Treatment and Management | No | N/A | No | N/A | Yes | Screening, Referral | No | N/A | No | Yes |
| Evaluating the role of levels of exposure to a task shared depression counselling intervention led by behavioural health counsellors: outcome and process evaluation | Selohilwe | 2019 | South Africa | Depression | Yes | Diagnosis, Referral | No | N/A | Yes | Diagnosis, Referral | No | N/A | Yes | Education and Counselling, Referral, Appointment Reminders | No | Yes |
| Experience of a 'Screen and treat' program for secondary prevention of cervical cancer in Uttar Pradesh, India | Shikha | 2020 | India | cervical cancer | Yes | Treatment and management | Yes | Treatment and management | No | N/A | Yes | Education and Counselling, Other - mobilize clients | No | N/A | No | Yes |
| Implementation of a community-based hypertension control program in Matanzas, Cuba | Valdes Gonzalez | 2019 | Cuba | hypertension | Yes | Diagnosis, Screening, Treatment and Management | No | N/A | Yes | Diagnosis, Screening | No | N/A | Yes | N/A | No | Yes |
| Evaluation of the "Take Five School": an education programme for people with Type 2 Diabetes in the Western Cape, South Africa | Van der Does | 2013 | South Africa | Type 2 Diabetes | Yes | Not specified | Yes | Not specified | No | N/A | No | N/A | No | N/A | No | Yes |

| | | | | | | | | | | | | | | | | |
|---|---|---|---|---|---|---|---|---|---|---|---|---|---|---|---|---|
| Community Health Workers Improve Linkage to Hypertension Care in Western Kenya | Vedanthan | 2019 | Kenya | Hypertension | Yes | Not specified | No | N/A | No | N/A | No | Yes | Not specified | N/A | No | Yes |
| An integrated program with home blood-pressure monitoring and village health volunteers for treating poorly controlled hypertension at the primary care level in an urban community of Thailand | Visanuyothin | 2018 | Thailand | hypertension | Yes | N/A | No | N/A | No | N/A | No | N/A | No | N/A | No | Yes |
| A model of translational research for diabetes prevention in low and middle-income countries: The Diabetes Community Lifestyle Improvement Program (D-CLIP) trial | Weber | 2012 | India | Diabetes Mellitus | Yes | Treatment and Management | Yes | Not specified | No | N/A | No | N/A | Yes | Education and Counselling, Other - physical activity | No | Yes |
| Impact of a Novel community-based lifestyle intervention program on type 2 diabetes and cardiovascular risk in a resource-poor setting in the Dominican Republic | West-Pollak | 2014 | Dominican Republic | Type 2 diabetes | Yes | Not specified | Yes | Not specified | No | N/A | No | N/A | Yes | Education and Counselling, Others- devised individualized lifestyle modification plans, physical activity, diet | No | Yes |
| ¡Si, Yo Puedo Vivir Sano con Diabetes! A Self-Management Randomized Controlled Pilot Trial for Low-Income Adults with Type 2 Diabetes in Mexico City | Whittemore | 2020 | Mexico | diabetes | No | N/A | No | N/A | Yes | Not specified | No | N/A | No | N/A | No | Yes |
| Effectiveness of a Multidisciplinary Approach Intervention to Improve Blood Pressure Control Among Elderly Hypertensive Patients in Rural Thailand: A Quasi-Experimental Study | Woodham | 2020 | Thailand | hypertension | Yes | N/A | No | N/A | Yes | Not specified | No | N/A | Yes | Education and Counselling | No | Yes |
| Educational program to improve hypertension kNowledge by a community pharmacist in a rural district in Indonesia | Wulandari | 2021 | Indonesia | Hypertension | Yes | N/A | No | N/A | No | N/A | No | N/A | No | N/A | No | Yes |
| Effect of group cognitive behavioural therapy on psychological stress and blood glucose in people with type 2 diabetes mellitus: A community-based cluster randomized controlled trial in China | Xu | 2021 | China | Type 2 diabetes mellitus | Yes | Treatment and Management, Education and Counselling, Follow-ups | No | N/A | No | N/A | No | N/A | No | N/A | No | No |
| Peer Support for Diabetes Management in Primary Care and Community Settings in Anhui Province, China | Zhong | 2015 | China | diabetes | No | N/A | No | N/A | No | N/A | No | N/A | Yes | Education and Counselling | No | Yes |
| Development and evaluation of a nurse-led hypertension management model: A randomized controlled trial | Zhu | 2018 | China | Hypertension | Yes | Treatment and Management | No | N/A | Yes | Telephone Follow-Ups, Referral, Other - Home Visits | No | N/A | No | N/A | No | No |
| Process Evaluation of a Clustered Randomized Control Trial of a Comprehensive Intervention to Reduce the Risk of Cardiovascular Events in Primary Health Care in Rural China | Zou | 2020 | China | CVDs | Yes | Diagnosis, Treatment and Management, Education and Counselling, Telephone follow-ups, Other - home visits. | No | N/A | No | N/A | No | N/A | No | N/A | No | No |
